# Synthesis of phthalazine-based derivatives as selective anti-breast cancer agents through EGFR-mediated apoptosis: in vitro and in silico studies

**DOI:** 10.1186/s13065-023-00995-2

**Published:** 2023-07-27

**Authors:** Sara M. Emam, Samir M. El Rayes, Ibrahim A. I. Ali, Hamdy A. Soliman, Mohamed S. Nafie

**Affiliations:** Department of Chemistry, Faculty of Science, Suez Canal University, Ismailia, 41522 Egypt

**Keywords:** Phthalazine-based, Selective anti-breast, EGFR-mediated, Apoptosis

## Abstract

The parent 2-(4-benzyl-1-oxophthalazin-2(1*H*)-yl)-acetohydrazide **(4)** has twenty-nine compounds. The starting material for their corresponding mono, dipeptides and reactions with active methylene compounds were produced by chemoselective *N*-alkylation of 4-Benzyl-2*H*-phthalazin-1-one **(2)** with ethyl chloroacetate to afford (4-benzyl-1-oxo-1*H*-phthalazin-2-yl) methyl acetate **(3)**. The ester **3** was hydrazinolyzed to give hydrazide **4**, then azide **5** coupled with amino acid ester hydrochloride and/or amines to produce several monopeptides, then the methyl (2-(4-benzyl-1-oxophthalazin-2(1*H*)-yl) acetyl) glycinate **(7a)** was hydrazinolyzed to produce corresponding hydrazide 2-(4-benzyl-1-oxophthalazin-2(1*H*)-yl)-*N*-(2-hydrazineyl-2-oxo ethyl) acetamide **(8a)**. The hydrazide **8a** under azide coupling method was coupled with amino acid ester hydrochloride and/or amines to produce several dipeptides, and the hydrazide **8a** was also condensed and/or cyclized with several carbonyl compounds. The cytotoxicity of the synthesized compounds was tested using MTT assay, as well as apoptosis-induction through EGFR inhibition. Compounds **11d**, **12c** and **12d** exhibited potent cytotoxic activities with IC_50_ values of 0.92, 1.89 and 0.57 μM against MDA-MB-231 cells compared to Erlotinib (IC_50_ = 1.02 μM). Interestingly compound **12d** exhibited promising potent EGFR inhibition with an IC_50_ value 21.4 nM compared to Erlotinib (IC_50_ = 80 nM). For apoptosis, compound **12d** induced apoptosis in MDA-MB-231 cells by 64.4-fold (42.5% compared to 0.66 for the control); hence, this compound may serve as a potential target-oriented anti-breast cancer agent. These results agreed with the molecular docking studies that highlighted the binding disposition of compound **12d** towards EGFR protein. Hence, compound **12d** may serve as a potential and selective anti-breast cancer agent.

**Supplementary Information:**

The online version contains supplementary material available at 10.1186/s13065-023-00995-2.

## Introduction

Cancer has been considered one of the major issues of concern, most especially for the public health system globally, which has been a leading cause of death worldwide in the last decade [1]. It is an abnormal development of cells that promulgates through the splitting of unrestricted cells, which shifts the controlled mechanisms of cell proliferation and differentiation associated with a high mortality rate [2]. Epidemiological studies revealed that cancer accounts for one of every five deaths, and it is estimated that the annual number of deaths due to cancers will increase from 7.6 million in 2008 to 13 million in 2030 [3]. Chemotherapy is one of the most effective approaches used to treat solid as well as hematological tumors [4, 5].Cancer chemotherapy has been developed for molecular therapeutics, which are more selective and not associated with the toxicity of conventional cytotoxic drugs [6].

After over half a century of chemotherapy research and despite the advancement in the knowledge of biochemical processes associated with carcinogenesis, the successful treatment of cancer remains a significant challenge because of the general toxicity associated with the clinical use of traditional cancer chemotherapeutic agents and because of some factors that include limitations of animal models, tumor diversity, drug resistance and the side effects assigned for therapy [7]. Therefore, anticancer drug research is never ending with obtaining lower toxicity and more selectivity products for tumor cells. There is an urgent need to give much attention to researchers in pharmaceuticals, medicine, and medicinal chemistry to design and modify the drug to fulfill more potent and effective therapies.

Epidermal growth factor receptor (EGFR) is a type of membrane-bound tyrosine kinase receptor which addicted to the treatment of cancer [8]. EGFR plays a vital role in numerous processes that affect tumor growth and progression, including proliferation, differentiation, angiogenesis, inhibition of apoptosis, and invasiveness [9]. The expression of a specific receptor tyrosine kinase on the cell surface increases the incidence of receptor dimerization. It leads to uncontrolled cell proliferation and tumor formation, which has been shown for EGFR to occur in breast, colon, ovarian, and pancreatic cancer cells [10]. Currently, large numbers of epidermal growth factor receptor inhibitors are approved, including gefitinib, erlotinib, lapatinib, vandetanib, etc. Amin [11] has reported a series of phthalazine derivatives as epidermal growth factor receptors.

Some phthalazine derivatives have significant applications in clinical medicine due to their pronounced activities as antitumor agents [12–15]. Hydrazides constitute an important class of compounds for new drug development as they contain H-bond donor/acceptors that can form H-bonds with their recepients inside the target-protein activie sites [16].^.^ Previous literacture [17–20] showed that phthalazine-based hydrazide derivatives represented a promising scaffold for kinase-targted anticancer agents, e.g. EGFR.

**Fig. 1:**
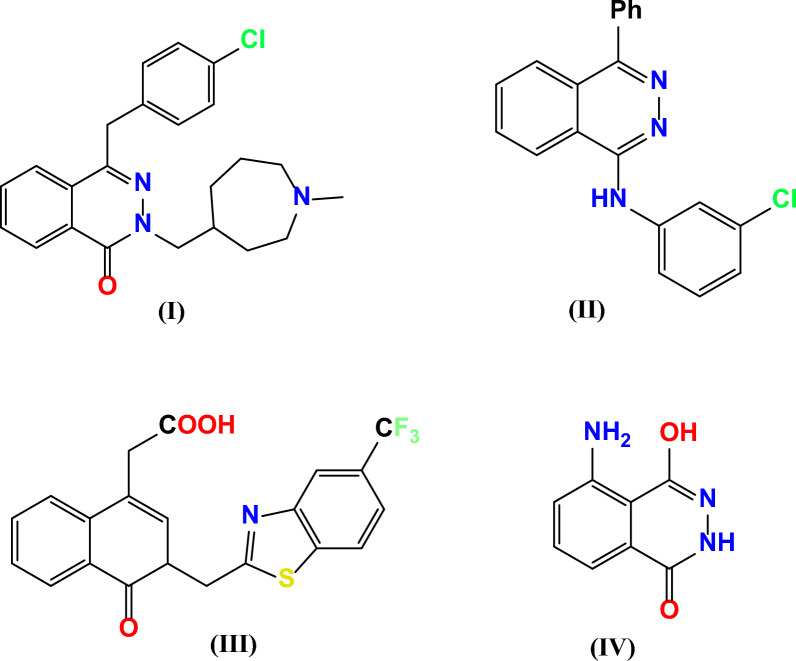
Some phthalazine-based derivatives

In Fig. 1, the phthalazine derivative azelastine **(I)** is an antihistamine used in the treatment of allergic rhinitis [21]. Potent agents are more selective inhibitors of the cGMP-inhibited phosphor diesterase (PDE) and can be represented by phthalazine derivatives like MY5445 **(II)** [22–25]. Zopolrestat **(III)** is a phthalazinone derivative that has been in clinical trials; it inhibits aldose reductase and has potential use in the prevention of retinopathy, neuropathy, and cataract formation in diabetes [26]. The chemiluminescence reactions of luminol **(IV)** and related phthalazines have found analytical applications, particularly in biological systems where the inherent signal strength and low signal–noise ratio contribute to sensitivity [27, 28].

**Fig. 2:**
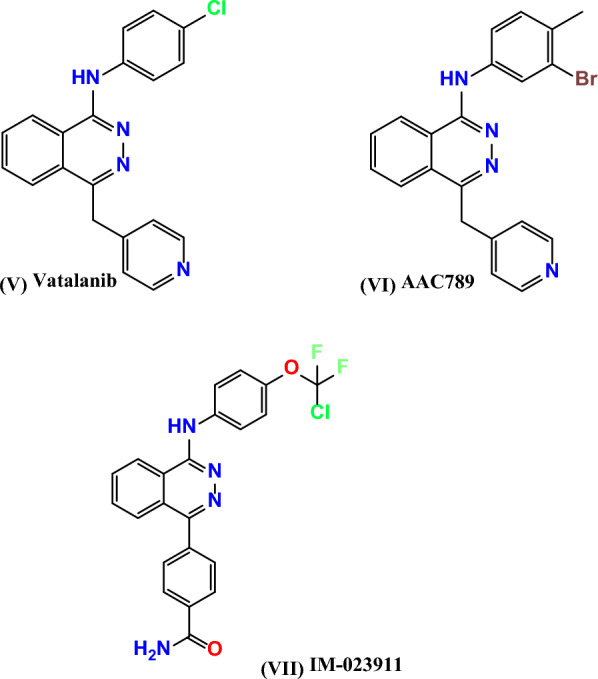
Phthalazine-based antitumor agents

Potent antitumor activity was addicted by many phthalazine-based compounds such as the anilino phthalazines, Vatalanib PTK787 **(V)** [12, 13, 29]. Vatalanib **(V)** inhibits VEGFR‐2 with IC_50_ value = 20 nM [30], and it is well absorbed orally and shows an in vivo antitumor activity against a panel of human tumor xenograft models; however, Vatalanib **(V)** is currently in phase III clinical trials for metastatic of colorectal cancer [31, 32]. In addition, some anilino phthalazines have been reported as potent inhibitors of VEGFR‐2, such as AAC789 **(VI)** and IM‐023911 **(VII)** with IC_50_ = 20 and 48 nM, respectively [33–38], as shown in Fig. 2.

Hence, the EGFR & VEGFR-2 inhibitory signaling pathway has become a crucial strategy for the identification and development of novel therapeutics for a variety of human malignancies for the treatment of cancer trauma [39]. So, we herein report the synthesis of new series of phthalazine derivatives aiming to obtain potent EGFR inhibitors with good anticancer activity.

## Results and discussion

### Chemistry

Most recently Samir El-Rayes et al. [40–44] reported early that, how can control on chemoselective alkylation in both amides and thioamides. As extension of these studies, we decided to apply these findings to structure modification of 4-benzyl-2*H* -phthalazin-1-one **(2)** as our model heterocyclic amide. The alkylation reaction of the model ambident nucleophile **2** with ethyl chloroacetate in Acetone/DMF mixture solution (1:1) in the presence of anhydrous K_2_CO_3_ under reflux condition for 20 h afforded (4-benzyl-1-oxo-1*H* -phthalazin-2-yl) methyl acetate **(3)** as a single *N*-substituted product.

The alkylation reaction proceeds depending on the behavior towards electrophiles according to reaction control points such as basicity and neucleophilicity of both N and O atoms. This reaction occurs selectively on N atom rather than on O atom or even in competition reaction at both atoms. The obtained chemoselective *N*- alkylation reaction may be well explained by counting on the interaction between HOMO at the nitrogen atom of the ambident nucleophile with high energy and the LUMO of the electrophile with low energy, leading to a narrow energy gap and high reactivity to finally give *N*-alkylation. This result was deduced on the premise of Pearson’s hard soft-acid base principle.

**Scheme 1:**
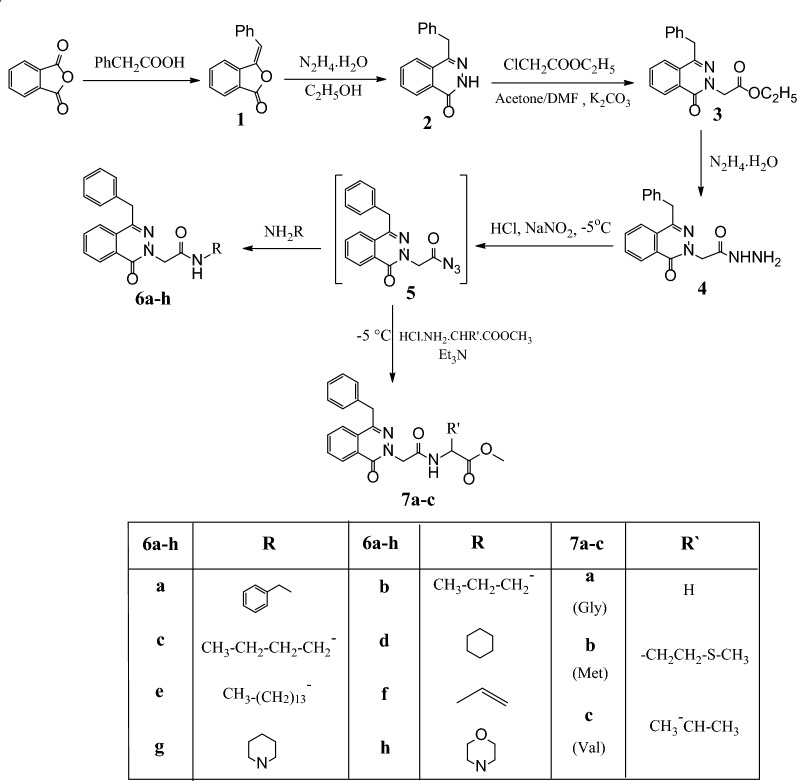
Preparation of 2-(4-benzyl-1-oxophthalazin-2(1*H*)-yl)-*N*-subsitituted derivatives **6a–h** and **7a–c**

Hydrazinolysis of ester **3** in ethanol via reaction with hydrazine hydrate under reflux for 6 h to produce the 2-(4-benzyl-1-oxophthalazin-2(1*H*)-yl)-acetohydrazide **(4)** in 90% yield which used as a precursor for the preparation of novel phthalazinone derivatives with potent importance in biological activity (Scheme 1).

The chemoselectivity alkylation occurs on the N-atom not the O isomer which prove that the N atom in present system is stronger neucleophile more than Oxygen, so this reaction is N-regioselective and this can be dedicated by the structure identification using ^1^H and ^13^C-NMR spectroscopy.

The characteristic 1H-NMR spectral peaks for the hydrazide **4** gave signals at δ 3.60 for NH_2_, 4.32 for CH_2_ph, 4.82 for NHCH_2_, 9.02 for NH and (7.18–8.43) for nine aromatic protons [45].

The hydrazide **4** is a superb forerunner for the structural adjustment of phthalazine subordinates by a connection of another amino acid through a peptide bond via azide coupling strategy, which is a well-known strategy in peptide synthesis having the advantage of diminishing the degree of racemization beside absence of any interferometer side products.

The azide **5** was prepared from the reaction between hydrazide **4** with NaNO_2_/HCl mixture in water for 1 h at − 5 °C that was extracted with ethyl acetate. The produced azide was progressively added to amines to give amide derivatives **6a–h** (Scheme 1).

The chemical structure of the synthesized 2-(4-benzyl-1-oxophthalazin-2(1*H*)-yl)-*N*-propyl acetamide **(6b)** was elucidated via different analysis methods for example the ^1^H-NMR that give characteristic protons at δ (7.15–8.38) nine protons of Ar–H, 6.16 broad signal for NH, 4.83 singlet peak for CH_2_CO, 4.23 singlet peak for CH_2_ph, 3.14–3.19 quartet peak of CH_2_NH, sextet and triplet peaks at 1.41–1.46 & 0.79–0.83 for CH_2_ and CH_3_ of propyl molecule respectively and the ^13^C-NMR spectrum has signals at 167.46 and 159.73 for two C=O groups and peaks at 55.33, 41.30, 38.83, 22.70 and 11.17 ppm for CH_2_CO, CH_2_NH,CH_2_ph, CH_2_, CH_3_ groups respectively by the addition to 13 aromatic carbons at (125.38–146.32) ppm.

The reaction of amino acid methyl ester hydrochloride such as glycine, methionine, and valine in the presence of triethyl amine at − 5 °C for 1 h to give the methyl-3-[2-(1,4-dioxo-3-phenyl-3,4-dihydro-1*H*-phthalazine-2-yl)-acetylamino]alkanoate**7a–c** (Scheme 1).

The glycine methyl ester of 4-benzyl-1(2*H*)-phthalazinone **7a** has the ^1^H-NMR spectrum of characteristic signals at δ 6.71 broad signal for NH molecule, 3.64 singlet peak of OCH_3_, 4.00–4.01 doublet peak of CH_2_NH, 4.23 and 4.90 ppm singlet peaks for CH_2_ph & CH_2_CO respectively and the ^13^C-NMR spectrum has signals at 169.99, 167.72 and 159.78 for three C=O groups, 54.80, 52.27, 41.29 and 38.91 ppm for CH_2_CO, OCH_3_, CH_2_NH and CH_2_ph groups respectively.

**Scheme 2:**
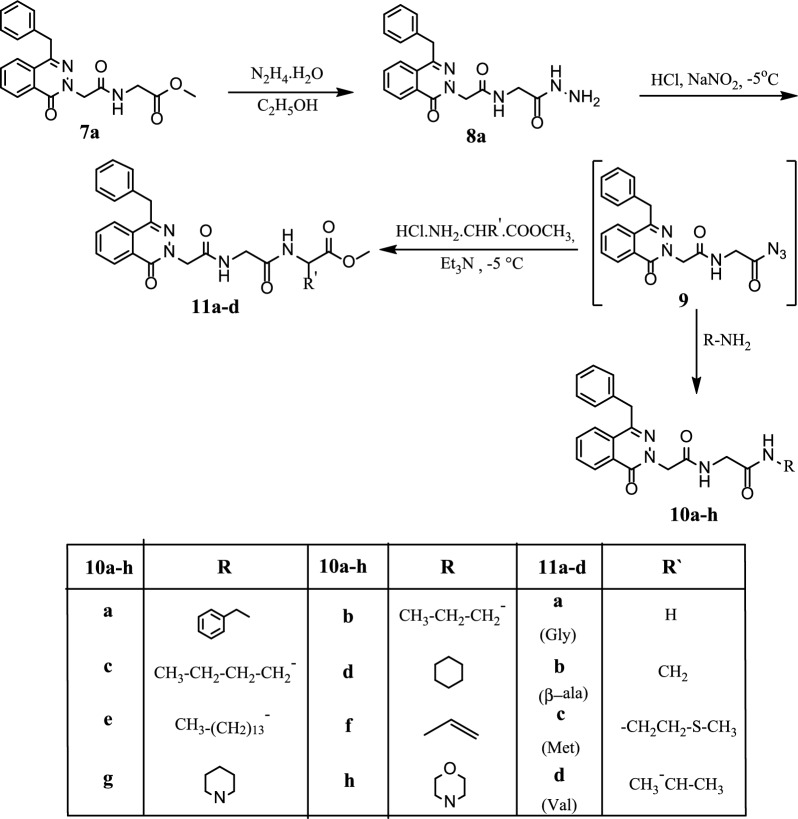
Synthesis of (4-benzyl-1-oxophthalazin-2(1*H*)-yl)-*N*-(2-oxo-2-(alkyl amino) ethyl) acetamides **10a–h** & alkanoates **11a–d**

The ester **7a** was considered a key intermediate for chemical structure modification of phthalazinone nucleus. The ester **7a** underwent hydrazinolysis via reflux with hydrazine hydrate in ethanol to produce the corresponding hydrazide **8a** as in Scheme 2.

The structure of starting hydrazide 2-(4-benzyl-1-oxophthalazin-2(1*H*)-yl)-*N*-(2-hydrazineyl-2-oxo ethyl)acetamide **(8a)** was elucidated by various analysis like ^1^H-NMR which give characteristic peaks for protons at δ 9.02 & 7.91–7.93 broad signals for 2 NH, 4.87 singlet peak for CH_2_CO, 4.32 singlet peak for CH_2_ph, 4.23 doublet peak of CH_2_NH and doublet peak at 3.74–3.75 for NH_2_ and the ^13^C-NMR spectrum has signals at 168.38, 167.73, 159.08 for three C=O groups and peaks at 53.98, 41.43, 38.13 ppm for CH_2_CO, CH_2_NH and CH_2_ph respectively.

Under azide coupling condition, 2-(4-benzyl-1-oxophthalazin-2(1*H*)-yl)-*N*-(2-hydrazineyl-2-oxoethyl)acetamide **(8a)** was treated with a mixture of sodium nitrite and HCl solution in water to give its corresponding azide solution which further reacted with different amines like benzyl, n-propyl, n-butyl, cyclohexyl, tetra decyl, allyl, pepridine and morphline amines to obtain *N*-substituted-2-(4-benzyl-1-oxophthalazin-2(1*H*)-yl)-2-oxoethyl) acetamides **10a–h**, as in Scheme 2.

The chemical structure of 2-(4-benzyl-1-oxophthalazin-2(1*H*)-yl)-*N*-(2-(butyl amino)-2-oxoethyl) acetamide **(10c)** was interpreted by ^1^H-NMR analysis including two broad singlet peaks at δ 6.76 and 6.50 for NH, 4.94 singlet peak of NCH_2_CO, 4.34 singlet peak of CH_2_ph, 3.99 doublet peak of NHCH_2_CO, 3.24–3.29 quartet peak for NHCH_2_CH_2_, 1.48–1.54 quintet peak for CH_2_CH_2_CH_2_, 1.32–1.37 sextet peak of CH_2_CH_2_CH_3_ and 0.90–0.93 triplet peak of CH_3_ and the ^13^C-NMR spectrum has signals at 168.34, 167.85 and 159.94 for three C=O groups, 55.80, 43.43, 39.41, 38.92, 31.41, 20.01 and 13.67 ppm for NCH_2_CO, NHCH_2_CO, NHCH_2_CH_2_, CH_2_ph CH_2_CH_2_CH_2_, CH_2_CH_2_CH_3_ and CH_3_ groups respectively.

The azide was coupled with different amino acid methyl esters such as glycine, β-alanine, methionine and valine in the presence of triethyl amine affording dipeptides methyl-[2-(4-benzyl-1-oxo-1*H* -phthalazin-2-yl)-acetylamino]alkanoates **11a–d** in reasonable yield (Scheme 2).

The structure of methyl 3-(2-(2-(4-benzyl-1-oxophthalazin-2(1*H*)-yl) acetamido acetamido) propanoate **(11b)** was interpreted by various analysis method including ^1^H-NMR analysis that noticed characteristic peaks: two broad singlet peaks at δ 6.88 for NH, 4.95 singlet peak of NCH_2_CO, 4.34 singlet peak of CH_2_ph, 3.97–3.98 doublet peak of NHCH_2_CO, 3.67 singlet peak of OCH_3_, 3.54–3.55 quartet peak for NHCH_2_CH_2_CO and 2.56–2.59 triplet peak for NHCH_2_CH_2_CO and the ^13^C-NMR spectrum has peaks at 172.50, 168.57, 167.88 and 159.93 of four C=O, 55.59, 51.73, 43.25, 38.91, 35.18 and 33.71 ppm for NCH_2_CO, OCH_3_, NHCH_2_CO, CH_2_ph, NHCH_2_CH_2_CO and NHCH_2_CH_2_CO respectively.

**Scheme 3:**
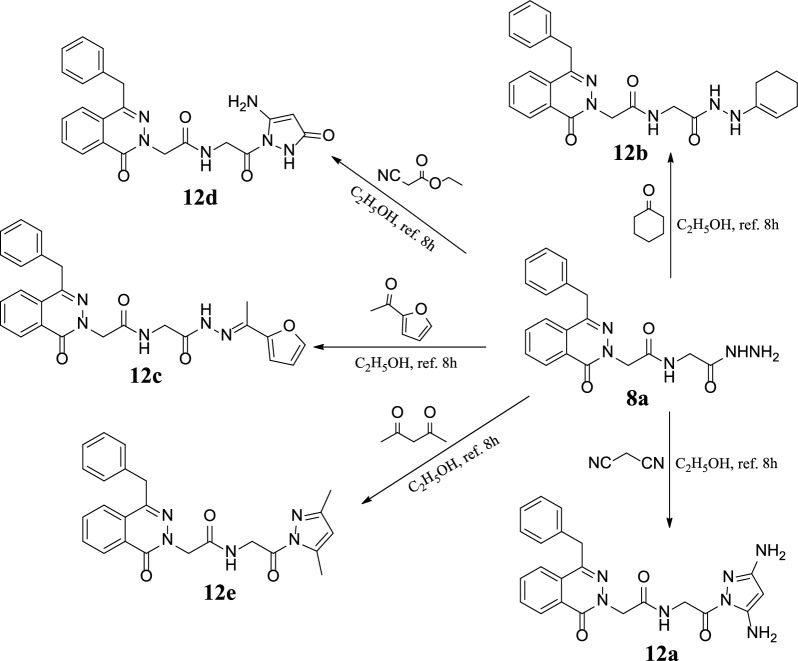
Synthesis of some derivatives of phthalazinone **12a–e**

Condensation of the hydrazide 2-(4-benzyl-1-oxophthalazin-2(1*H*)-yl)-*N*-(2-hydrazineyl-2-oxoethyl)acetamide **(8a)** with active methylene compounds such as malononitrile, ethyl cyano acetate and acetyl acetone in ethanol under reflux to obtain novel derivatives of phthalazinone **12a, 12d** and **12e** respectively in reasonable yield. Similarly, reaction of hydrazide **8a** with ketones such as cyclohexanone and 2-furyl methyl ketone gave the corresponding hydrazones **12b** and **12c** respectively as shown in Scheme 3.

The chemical structure of 2-(4-benzyl-1-oxophthalazin-2(1*H*)-yl)-*N*-2-(3,5-diamino-1*H*-pyrazol-1-yl)-2-oxoethyl)acetamide **(12a)** was elucidated using various analysis methods for example ^1^H-NMR which gave signals at δ 9.02 broad signal for NHCH_2_CO, 7.10–7.12 singlet peak for the proton of pyrazole ring NH_2_-C=CH-C-NH_2_, 5.46–5.47 doublet peak for NHCH_2_CO, 4.86 singlet peak of NCH_2_CO, 4.26 singlet peak of CH_2_ph and 4.32 & 3.52 two broad singlet peaks for 2 NH_2_ molecules on pyrazole ring and the ^13^C-NMR spectrum has peaks at 171.42, 168.34 and 159.21 of three C=O, 148.14 & 148.36 peaks of carbons of pyrazole ring 2 C-NH_2_, 110.35 characteristic peak for carbon atom of NH_2_-C=CH-C-NH_2_, 55.28, 42.82 and 38.64 ppm for NCH_2_CO, NHCH_2_CO and CH_2_ph respectively.

## Biological investigation

### Cytotoxicity against breast cancer cells

**Fig. 3:**
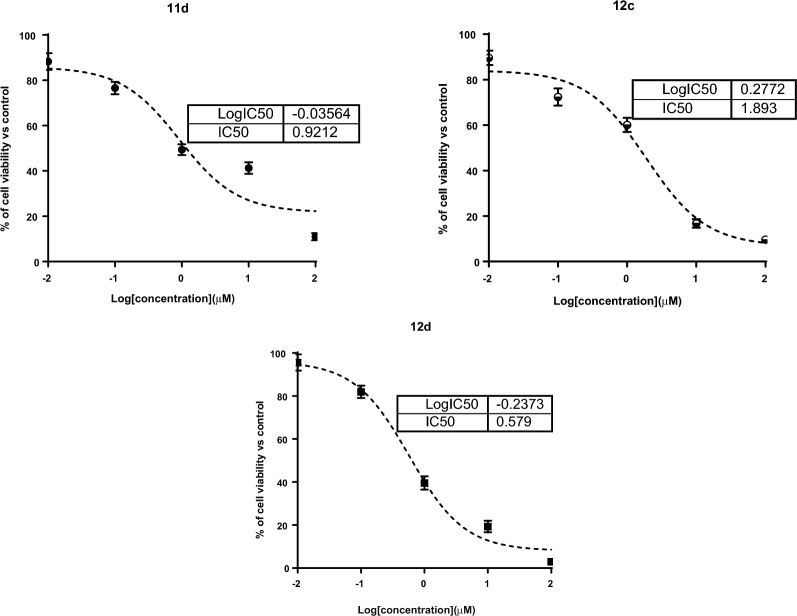
Percentage of cell growth inhibition versus concentrations of compounds **11d, 12c** and **12d** against caner MCF-7 and MDA-MB-231 cells using MTT assay using serial concentration range of 100 µM to 0.01 µM at incubation time of 48 h. Values are expressed as Mean ± SD of three independent values

The synthesized compounds were investigated for their cytotoxicity against breast MCF-7 and MDA-MB-231 cancer cell lines; IC_50_ values were summarized in Table 1. As seen in the results, interestingly, compounds **11d, 12c,** and **12d** exhibited potent cytotoxic activities against MCF-7 cells with IC_50_ values of 2.1, 1.4, and 1.9 μM, and against MDA-MB-231cells with potent IC_50_ values of 0.92, 1.89 and 0.57 μM, respectively, compared to erlotinib as the reference drug with IC_50_ values of 1.32 and 1.0 μM. As seen in Fig. 3, compound **12d** caused cell MDA-MB-231 cell growth inhibition by 98.2% at the highest concentration. Additionally, compounds **11d**, **12c,** and **12d** exhibited safe cytotoxicity against normal breast cells MCF-10A, having a percentage of cell viability of 11%, 9.6%, and 3%, respectively, at the highest concentration with IC_50_ values of 39.4, 43.6, and 41.6 μM. Based on these results, compound **12d** was worthy of further testing against EGFR enzymatic targets and the mechanism of cell death in MDA-MB-231 cells.

**Table 1:** Cytotoxicity of the synthesized derivatives against MCF-7, MBA-MB-231 and MCF-10A cells

Compound	IC_50_ (μM) ± SD*		
	MCF-7	MDA-MB-231	MCF-10A
**6f**	23.1 ± 0.94	≥ 50	NT
**6a**	18.6 ± 0.69	19.8 ± 0.74	NT
**6 h**	17.3 ± 0.31	21.3 ± 0.64	NT
**7a**	≥ 50	26.5 ± 0.79	NT
**7c**	12.4 ± 0.34	≥ 50	NT
**8a**	31.2 ± 1.01	1.34 ± 0.13	NT
**10a**	≥ 50	6.4 ± 0.18	NT
**10f**	15.4 ± 0.28	8.6 ± 0.1	NT
**10h**	16.4 ± 0.31	2.3 ± 0.1	NT
**11a**	11.3 ± 0.29	7.2 ± 0.3	NT
**11d**	2.1 ± 0.01	0.92 ± 0.01	39.4 ± 1.8
**12c**	1.4 ± 0.05	1.89 ± 0.04	43.6 ± 1.9
**12d**	1.9 ± 0.01	0.57 ± 0.09	41.6 ± 1.8
**Erlotinib**	1.32 ± 0.04	1.02 ± 0.1	30.9 ± 1.8

“*IC_50_ values were calculated as the average of three independent trials using a dose–response curve in GraphPad prism”. *NT*  not tested

### Structure–activity relationship (SAR)

**Fig. 4:**
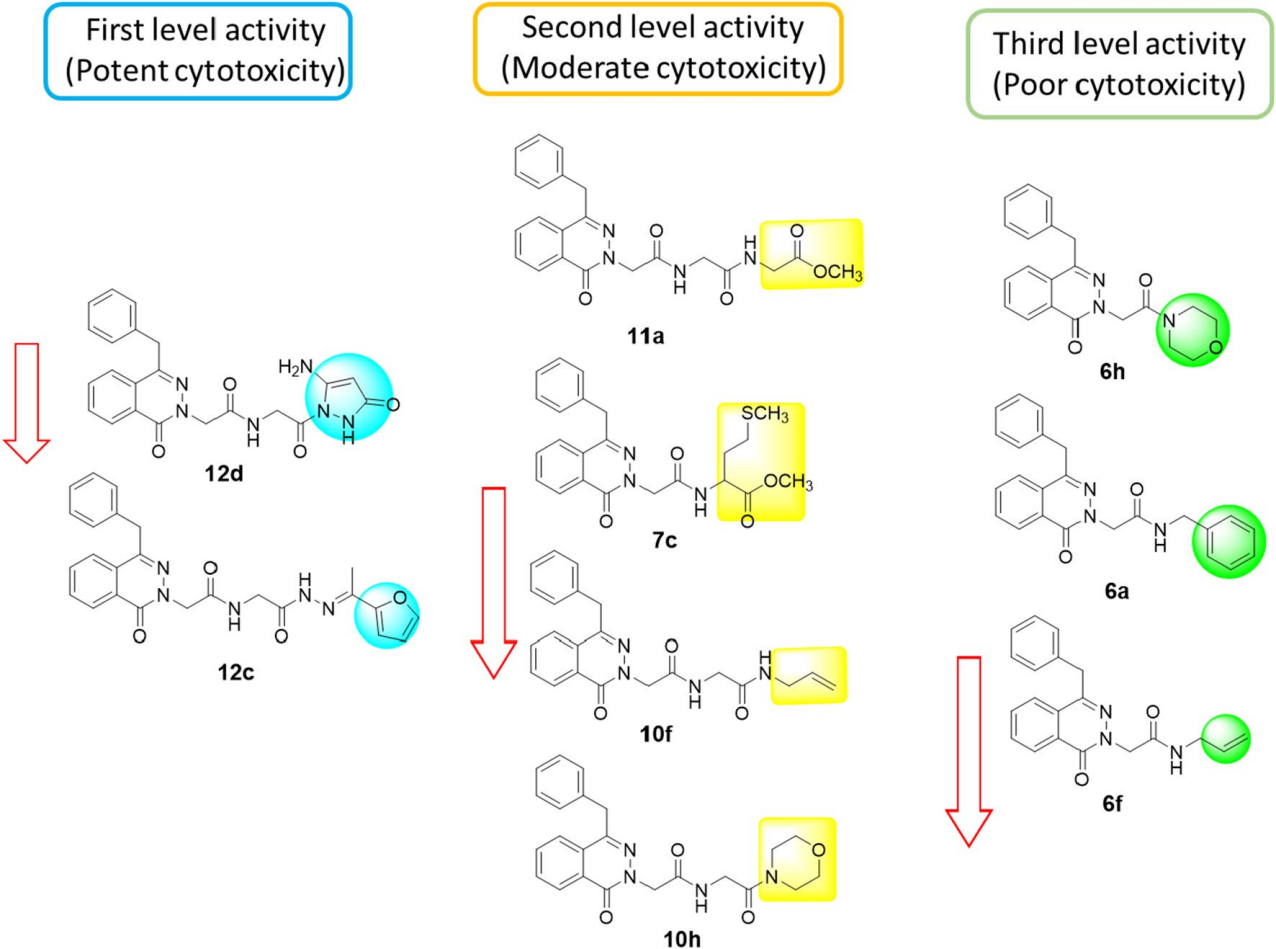
Highlighted substituents anchored on the pharmacophore with promising cytotoxic activities for investigated compounds

Based on the cytotoxicity results of the investigated compounds as summarized previously in Table 1, compounds **12d** and **12c** were the first order activity of potent cytotoxicicty (IC_50_ ≤ 2.5 μM), and compounds **11a**, **7c**, **10f**, and **10 h** with second order activity of moderate cytotoxicity (IC_50_ ≤ 20 μM), while compounds **6 h**, **6a** and **6f** with poor cytotoxicity (IC_50_ ≥ 20 μM). As summarized in Fig. 4, highlighted substituents caused variance in activity. Hence, compound **12d** was worthy to be further investigated for the effective target and cell death mechanism.

### EGFR enzyme inhibition

Three compounds with potent cytotoxicity **11d**, **12c,** and **12d** were tested for their inhibition against VEGFR2; interestingly, as seen in Table 2, compound **12d** exhibited promising EGFR enzyme inhibition with IC_50_ values of 21.4 nM with 97.6% inhibition compared to erlotinib with standard EGFR inhibition with IC_50_ value of 80 nM (inhibition 93.9%). Additionally, compounds **11d** and **12c** exhibited promising EGFR inhibitory activities with IC_50_ values 79.6 and 65.4 nM, with enzyme inhibition by 92.9% and 96.2%, respectively.

**Table 2:** EGFR enzyme activity of compounds **11d**, **12c** and** 12d**

Compound	EGFR	
	% of inhibition at [10 µM]	IC_50_ [nM] ± SD*
**11d**	92.9 ± 2.08	79.6 ± 1.35
**12c**	96.2 ± 2.04	65.4 ± 1.12
**12d**	97.6 ± 2.49	21.4 ± 0.67
**Erlotinib**	**93.9** ± 2.68	80.1 ± 1.21

*Values are expressed as an average of three independent replicates. “IC_50_ values were calculated using sigmoidal non-linear regression curve fit of percentage inhibition against five concentrations of each compound”

### Apoptosis-induction activity

**Fig. 5:**
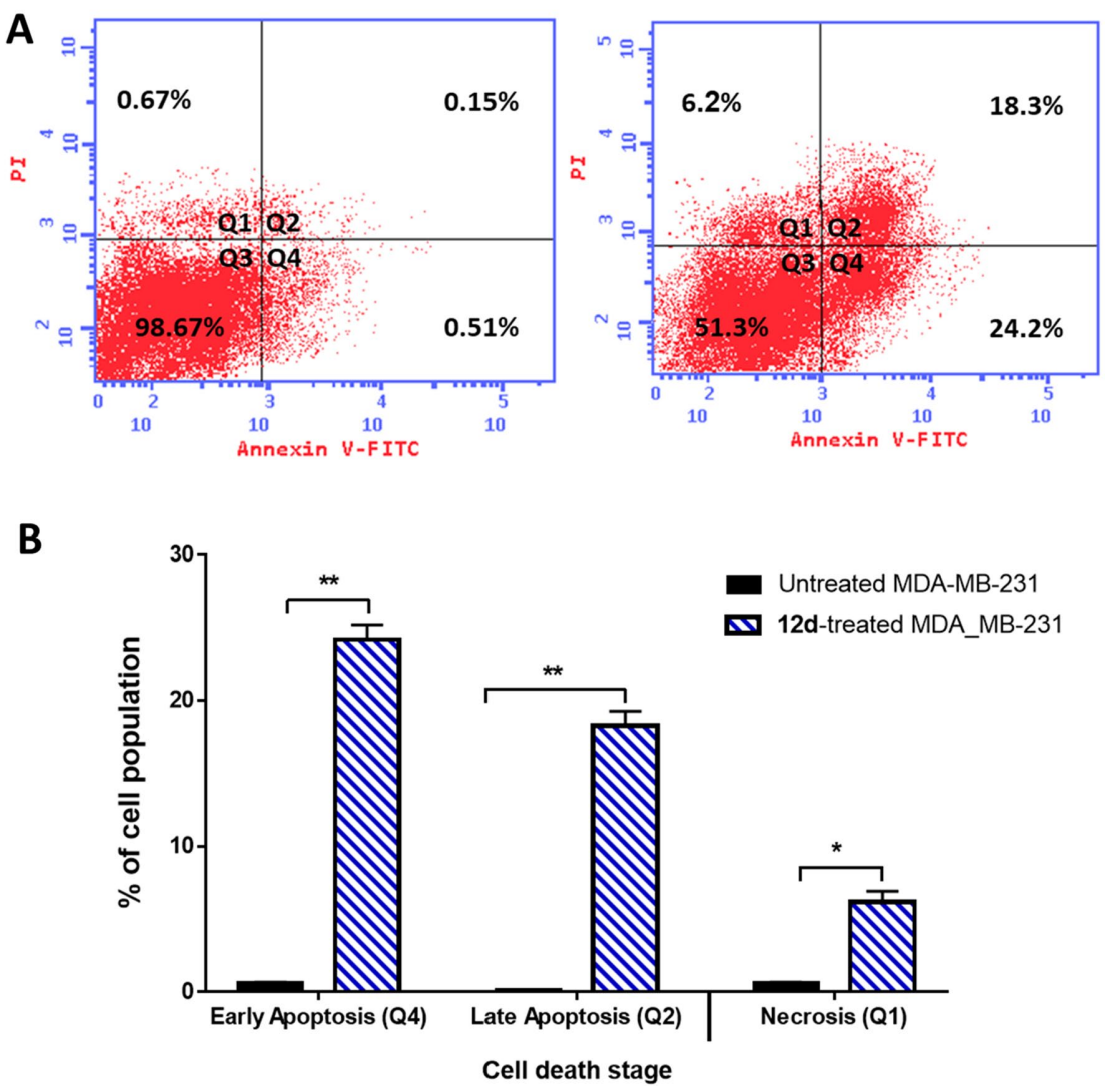
Flow cytometry analysis for apoptosis/necrosis assessment in the untreated and **12d**-treated MDA-MB-231 cells with the IC_50_ value of 0.57 μM for 48 h **A** Cytogram for Annexin V/PI staining. Quadrant charts show Q1 (necrotic cells, AV−/PI +), Q2 (late apoptotic cells, AV + /PI +), Q3 (normal cells, AV−/PI−), Q4 (early apoptotic cells, AV + /PI−). **B** Bar representation with cell percentage at each stage. Values are expressed as Mean ± SD of three independent trials “*(P ≤ 0.05), and **(P ≤ 0.001) are significantly different using the un-paired test in GraphPad prism”

Deregulation of apoptosis is a hallmark of all cancer cells, and the agents that activate apoptosis in cancer cells could be valuable anticancer therapeutics; breast cancer cell lines that hyper express the EGFR have been documented to undergo receptor-mediated apoptosis. MDA-MB-231 cancer cells were treated with compound **12d** (IC_50_ = 0.57 μM, 48 h) and were investigated for their apoptosis-inducing activity using Annexin V/PI staining. As seen in Fig. 5, compound **12d** significantly stimulated total apoptotic breast cancer cell death by 64.4-fold (42.5% compared to 0.66% for the control). It induced early apoptosis by 24.2% and late apoptosis by 18.3% compared to 0.66% and 0.15%, respectively, for the control. Moreover, it stimulated cell death by necrosis by 9.25-fold (6.2%, compared to 0.67% for the control).

These results of apoptosis-induction of phthalazine-based derivatives agreed with previous studies that exhibited promising cytotoxic activities as apoptotic agents through EGFR inhibition.

### Molecular docking studies

**Fig. 6:**
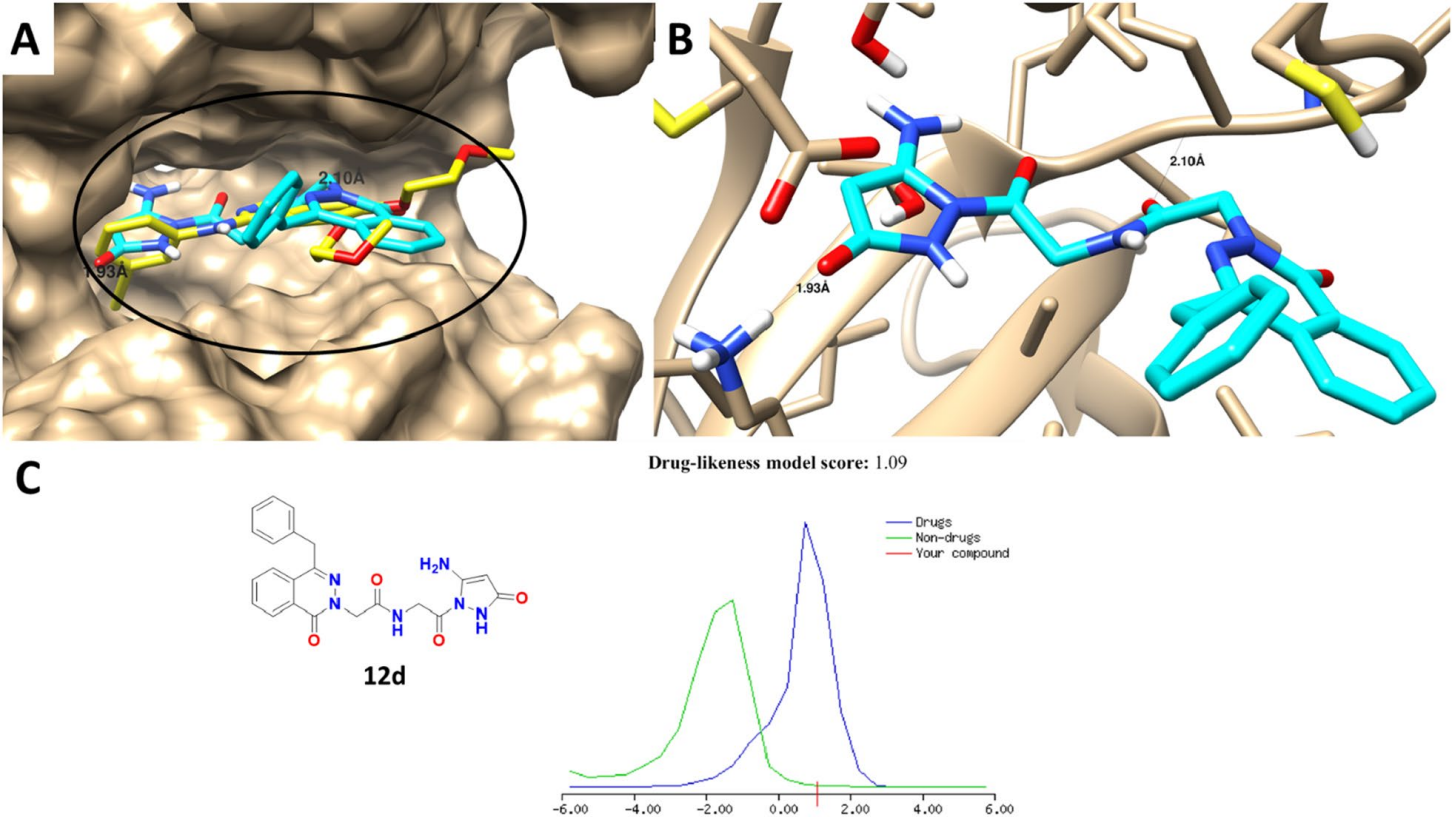
Binding disposition and molecular docking interactions of the docked compound **12d** (Cyan-colored) and the co-crystallized ligand (Yellow-colored). **A** Surface view and **B** Interactive view with ribbon presentation. **C** Drug-likeness properties of compound **12d** using MolSoft “The green color means non-drug like behavior and those fall under blue color area are considered as drug-like

Based on the promising EGFR inhibition activity of compound **12d**, it was screened for virtual binding towards EGFR protein using the molecular docking approach. As shown in Fig. 6, compound **12d** was docked inside EGFR protein with a binding energy of − 18.4 kcal/mol and formed one H-bond with Met 769, one H-bond with Lys 721, besides it formed the lipophilic interactions through phenyl groups with the lipophilic amino acids of Ala 719 and Leu 694. Hence, docking results indicated highlighted the virtual mechanism of binding of compound **12d** through the phthalazine moiety for interactions toward EGFR protein, which agreed with its promising experimental activity. Physiochemical properties and ADME pharmacokinetics revealed the drug-likeness score of 1.09, which obeys the Lipinsiki’s rule of five, having molecular weight = 432 g/mol, topological polar surface area (TPSA) = 144.8 A^2^, log (P) = 1.56, H-bond donor = 4, and H-bond acceptor = 5.

## Experimental

### Chemistry

#### General procedures

The purity of the synthesized compounds was checked by thin layer chromatography (TLC) technique was carried out on silica gel 60 F_254_ aluminum sheets (E. Merck, layer thickness 0.2 mm) in the following solvent system ethyl acetate/ petroleum ether (1:1) and ethyl acetate/ petroleum ether (2:1), the spots on thin layer plates were detected by UV lamp. The melting points were determined using a Buchi 510 melting-point system and are uncorrected. At the Micro Analytical Laboratory, Faculty of Science, Cairo University, Cairo, Egypt, element analyses were performed on a Flash EA-1112 apparatus. The nuclear magnetic resonance laboratory, Faculty of Science, Sohag University, Egypt, used a Bruker spectrometer running at 400 MHz to estimate 1H-NMR spectra.

The precursor (4-benzyl-1-oxo-1*H* -phthalazin-2-yl) methyl acetate **(3)** was prepared from 4-Benzyl-2H-phthalazin-1-one **(2)** according to the method described in Marzouk et al. [45] that was converted to the hydrazide molecule 2-(4-benzyl-1-oxophthalazin-2(1*H*)-yl)-acetohydrazide **(4)** [13].

##### General procedure for preparation of 2-(4-benzyl-1-oxophthalazin-2(1*H*)-yl)-*N*-alkyl acetamide 6a–h

A cold solution at (− 5 °C) of acetohydrazide **4** (3.08 g, 10 mmol) in acetic acid (60 mL) and hydrochloric acid (5N, 30 mL) was added portion wise under stirring to a cold solution (0 °C) of sodium nitrite (0.7 g, 0.01 mol) in water (30 mL). After stirring at the same temperature for 30 min, the in situ generated azide was extracted with cold ethyl acetate and washed successively with cold water and 5% Na_2_CO_3_.

After drying over anhydrous sodium sulphate, the azide was used without further purification in the next step. Amines (12 mmol) were added to the previously prepared cold dried solution of the azide. Afterwards, the mixture was kept 12 h in the refrigerator and then at room temperature for another 12 h. The reaction mixture was filtered and the filtrated solution washed with 0.1N HCl, 5% Na_2_CO_3_ and water then dried over anhydrous sodium sulphate, the solvent was evaporated in vacuum and the residue was crystallized from ethyl acetate-petroleum ether to give products **6a–h**.

##### Synthesis of 2-(4-benzyl-1-oxophthalazin-2(1H)-yl)-N-benzyl acetamide (6a)

White crystals (84%), m.p. 174–176 °C, ^1^H-NMR (400 MHz, CDCl_3_), (δ, ppm), (*J*, Hz): 8.42–8.44 (m, 1H, ArH); 7.70–7.76 (m, 3H, ArH); 7.27–7.30 (m, 9H, ArH); 7.22–7.25 (m, 1H, ArH); 6.62 (brs, 1H, NH); 4.99 (s, 2H, CH_2_CO); 4.50–4.51 (d, *J* = 5.6, 2H, CH_2_NH); 4.32 (s, 2H, CH_2_ph).^13^C-NMR: 167.47 (C=O); 159.75 (C=O); 146.39 (C-Ar); 138.02 (C-Ar); 137.51 (C-Ar); 133.22 (CH-Ar); 131.45 (CH-Ar); 129.45 (C-Ar); 128.74 (2 CH-Ar); 128.61 (2 CH-Ar); 128.39 (2 CH-Ar); 128.08 (C-Ar); 127.57 (2 CH-Ar);127.37 (2 CH-Ar); 126.78 (CH-Ar); 125.39 (CH-Ar); 55.16 (CH_2_CO); 43.56 (CH_2_NH); 38.88 (CH_2_ph).

MS (MALDI, positive mode, matrix DHB) m/z: 406.48 (M + Na)^+^. Elemental analysis: calculated for C_24_H_21_N_3_O_2_ (383.45): % C, 75.18; % H, 5.52; % N, 10.96. Found: % C, 75.20; % H, 5.53; % N, 10.92.

##### Synthesis of 2-(4-benzyl-1-oxophthalazin-2(1*H*)-yl)-*N*-propyl acetamide (6b)

White crystals (82%), m.p. 162–164 °C, ^1^H-NMR (400 MHz, CDCl_3_), (δ, ppm), (*J*, Hz): 8.36–8.38 (m, 1H, ArH); 7.63–7.68 (m, 3H, ArH); 7.19–7.21 (m, 4H, ArH); 7.12–7.15 (m, 1H, ArH); 6.16 (brs, 1H, NH); 4.83 (s, 2H, CH_2_CO); 4.23 (s, 2H, CH_2_ph); 3.14–3.19 (q, 2H, CH_2_NH); 1.41–1.46 (sextet, 2H, CH_2_); 0.79–0.83 (t, *J* = 7.2, 3H, CH_3_).^13^C-NMR: 167.46 (C=O); 159.73 (C=O); 146.32 (C-Ar); 137.56 (C-Ar); 133.21 (CH-Ar); 131.44 (CH-Ar); 129.44 (C-Ar); 128.72 (2 CH-Ar); 128.39 (2 CH-Ar); 128.11 (C-Ar); 127.38 (CH-Ar);126.79 (CH-Ar); 125.38 (CH-Ar); 55.33 (CH_2_CO); 41.30 (CH_2_NH); 38.83 (CH_2_ph); 22.70 (CH_2_);11.17 (CH_3_).

MS (MALDI, positive mode, matrix DHB) m/z: 358.43 (M + Na)^+^. Elemental analysis: calculated for C_20_H_21_N_3_O_2_ (335.41): % C, 71.62; % H, 6.31; % N, 12.53. Found: % C, 71.63; % H, 6.33; % N, 12.51.

##### Synthesis of 2-(4-benzyl-1-oxophthalazin-2(1*H*)-yl)-*N*-butyl acetamide (6c)

White crystals (85%), m.p. 170–172 °C, ^1^H-NMR (400 MHz, CDCl_3_), (δ, ppm), (*J*, Hz): 8.34–8.37 (m, 1H, ArH); 7.61–7.68 (m, 3H, ArH); 7.19–7.20 (m, 4H, ArH); 7.11–7.14 (m, 1H, ArH); 6.17 (brs, 1H, NH); 4.82 (s, 2H, CH_2_CO); 4.22 (s, 2H, CH_2_ph); 3.17–3.21 (q, 2H, CH_2_NH); 1.35–1.42 (qn, 2H, CH_2_); 1.20–1.28 (sextet, 2H, CH_2_); 0.78–0.82 (t, *J* = 7.2, 3H, CH_3_).^13^C-NMR: 167.49 (C=O); 159.79 (C=O); 146.43 (C-Ar); 137.55 (C-Ar); 133.34 (CH-Ar); 131.54 (CH-Ar); 129.39 (C-Ar); 128.78 (2 CH-Ar); 128.40 (2 CH-Ar); 128.02 (C-Ar); 127.36 (CH-Ar);126.84 (CH-Ar); 125.47 (CH-Ar); 55.33 (CH_2_CO); 39.39 (CH_2_NH); 38.89 (CH_2_ph); 31.54 (CH_2_); 20.02 (CH_2_);13.73 (CH_3_).

MS (MALDI, positive mode, matrix DHB) m/z: 372.46 (M + Na)^+^. Elemental analysis: calculated for C_21_H_23_N_3_O_2_ (349.43): % C, 72.18; % H, 6.63; % N, 12.03. Found: % C, 72.21; % H, 6.61; % N, 12.07.

##### Synthesis of 2-(4-benzyl-1-oxophthalazin-2(1*H*)-yl)-*N*-cyclohexyl acetamide (6d)

White crystals (86%), m.p. 158–160 °C, ^1^H-NMR (400 MHz, CDCl_3_), (δ, ppm), (*J*, Hz): 8.44–8.47 (m, 1H, ArH); 7.71–7.76 (m, 3H, ArH); 7.27–7.30 (m, 4H, ArH); 7.21–7.24 (m, 1H, ArH); 6.12 (brs, 1H, NH); 4.90 (s, 2H, CH_2_CO); 4.32 (s, 2H, CH_2_ph); 3.82 (m, 1H, CHNH); 1.11–1.89 (m, 10H, 5 CH_2_).^13^C-NMR: 166.58 (C=O); 159.73 (C=O); 146.35 (C-Ar); 137.58 (C-Ar); 133.31 (CH-Ar); 131.52 (CH-Ar); 129.37 (C-Ar); 128.78 (2 CH-Ar); 128.39 (2 CH-Ar); 128.04 (C-Ar); 127.41 (CH-Ar);126.83 (CH-Ar); 125.45 (CH-Ar); 55.25 (CH_2_CO); 48.40 (CHNH); 38.87 (CH_2_ph); 32.92 (2 CH_2_); 25.49 (CH_2_);24.72 (2 CH_2_).

MS (MALDI, positive mode, matrix DHB) m/z: 398.50 (M + Na)^+^. Elemental analysis: calculated for C_23_H_25_N_3_O_2_ (375.47): % C, 73.57; % H, 6.71; % N, 11.19. Found: % C, 73.55; % H, 6.75; % N, 11.24.

##### Synthesis of 2-(4-benzyl-1-oxophthalazin-2(1*H*)-yl)-*N*-tetradecyl acetamide (6e)

White crystals (82%), m.p. 126–128 °C, ^1^H-NMR (400 MHz, CDCl_3_), (δ, ppm), (*J*, Hz): 8.45–8.47 (m, 1H, ArH); 7.71–7.76 (m, 3H, ArH); 7.26–7.30 (m, 4H, ArH); 7.22–7.24 (m, 1H, ArH); 6.22 (brs, 1H, NH); 4.92 (s, 2H, CH_2_CO); 4.32 (s, 2H, CH_2_ph); 3.27 (q, 2H, CH_2_NH); 1.22–1.66 (m, 24H, 12 CH_2_); 0.87–0.91 (t, *J* = 7.2, 3H, CH_3_). ^13^C-NMR: 167.50 (C=O); 159.78 (C=O); 146.42 (C-Ar); 137.54 (C-Ar); 133.33 (CH-Ar); 131.53 (CH-Ar); 129.38 (C-Ar); 128.78 (2 CH-Ar); 128.40 (2 CH-Ar); 128.02 (C-Ar); 127.35 (CH-Ar); 126.84 (CH-Ar); 125.46 (CH-Ar); 55.32 (CH_2_CO); 39.71 (CH_2_NH); 38.90 (CH_2_ph); 31.93 (CH_2_); 29.66 (2 CH_2_);29.56 (2 CH_2_); 29.46 (2 CH_2_); 29.36 (2 CH_2_); 29.26 (CH_2_); 26.86 (CH_2_); 22.70 (CH_2_); 14.13 (CH_3_).

MS (MALDI, positive mode, matrix DHB) m/z: 512.68 (M + Na)^+^. Elemental analysis: calculated for C_31_H_43_N_3_O_2_ (489.70): % C, 76.03; % H, 8.85; % N, 8.58. Found: % C, 76.09; % H, 8.80; % N, 8.60.

##### Synthesis of 2-(4-benzyl-1-oxophthalazin-2(1*H*)-yl)-*N*-allyl acetamide (6f)

White crystals (87%), m.p. 164–166 °C, ^1^H-NMR (400 MHz, CDCl_3_), (δ, ppm), (*J*, Hz): 8.36–8.39 (m, 1H, ArH); 7.63–7.68 (m, 3H, ArH); 7.18–7.21 (m, 4H, ArH); 7.13–7.16 (m, 1H, ArH); 6.21 (brs, 1H, NH); 5.70–5.79 (m, 1H, CH=CH_2_); 5.01–5.12 (dd, *J* = 17.2, *J* = 13.2, *J* = 10.4, 2H, CH_2_=CH); 4.87 (s, 2H, CH_2_CO); 4.24 (s, 2H, CH_2_ph); 3.83–3.85 (t, *J* = 5.6, 2H, CH_2_NH). ^13^C-NMR: 167.47 (C=O); 159.82 (C=O); 146.57 (C-Ar); 137.49 (C-Ar); 133.78 (CH-Ar); 133.40 (CH=CH_2_); 131.60 (CH-Ar); 129.40 (C-Ar); 128.79 (2 CH-Ar); 128.42 (2 CH-Ar); 127.99 (C-Ar); 127.38 (CH-Ar);126.86 (CH-Ar); 125.49 (CH-Ar); 116.43 (CH=CH_2_); 55.25 (CH_2_CO); 41.92 (CH_2_NH); 38.90 (CH_2_ph).

MS (MALDI, positive mode, matrix DHB) m/z: 356.41 (M + Na)^+^. Elemental analysis: calculated for C_20_H_19_N_3_O_2_ (333.39): % C, 72.05; % H, 5.74; % N, 12.60. Found: % C, 72.01; % H, 5.77; % N, 12.54.

##### Synthesis of 4-benzyl-2-(2-oxo-2-(piperidin-1-yl) ethyl) phthalazin-1(2*H*)-one (6g)

White crystals (80%), m.p. 192–194 °C, ^1^H-NMR (400 MHz, CDCl_3_), (δ, ppm), (*J*, Hz): 8.36–8.37 (m, 1H, ArH); 7.60 (m, 3H, ArH); 7.20–7.21 (m, 4H, ArH); 7.12 (m, 1H, ArH); 5.01 (s, 2H, CH_2_CO); 4.23 (s, 2H, CH_2_ph); 3.53 (m, 2H, CH_2_N); 3.41 (m, 2H, CH_2_N); 1.53–1.60 (m, 6H, 3 CH_2_). ^13^C-NMR: 164.92 (C=O); 159.70 (C=O); 145.24 (C-Ar); 138.00 (C-Ar); 132.79 (CH-Ar); 130.98 (CH-Ar); 129.68 (C-Ar); 128.66 (2 CH-Ar); 128.38 (2 CH-Ar); 128.38 (C-Ar); 127.37 (CH-Ar);126.58 (CH-Ar); 125.24 (CH-Ar); 52.54 (CH_2_CO); 45.96 (CH_2_N); 43.29 (CH_2_N); 39.00 (CH_2_ph); 26.25 (CH_2_CH_2_N);25.38 (CH_2_CH_2_N); 24.47 (CH_2_CH_2_CH_2_N).

MS (MALDI, positive mode, matrix DHB) m/z: 384.44 (M + Na)^+^. Elemental analysis: calculated for C_22_H_23_N_3_O_2_ (361.45): % C, 73.11; % H, 6.41; % N, 11.63. Found: % C, 73.08; % H, 6.39; % N, 11.60.

##### Synthesis of 4-benzyl-2-(2-morpholino-2-oxoethyl) phthalazin-1(2*H*)-one (6h)

Off-white crystals (81%), m.p. 210–212 °C, ^1^H-NMR (400 MHz, CDCl_3_), (δ, ppm), (*J*, Hz): 8.44–8.46 (m, 1H, ArH); 7.68–7.73 (m, 3H, ArH); 7.29–7.30 (m, 4H, ArH); 7.20–7.23 (m, 1H, ArH); 5.10 (s, 2H, CH_2_CO); 4.32 (s, 2H, CH_2_ph); 3.59–3.75 (m, 8H, 4 CH_2_). ^13^C-NMR: 165.45 (C=O); 159.71 (C=O); 145.60 (C-Ar); 137.83 (C-Ar); 133.01 (CH-Ar); 131.19 (CH-Ar); 129.62 (C-Ar); 128.71 (2 CH-Ar); 128.36 (2 CH-Ar); 128.10 (C-Ar); 127.33 (CH-Ar);126.68 (CH-Ar); 125.36 (CH-Ar); 66.78 (CH_2_O); 66.40 (CH_2_O); 52.31 (CH_2_CO); 45.37 (CH_2_N); 42.41 (CH_2_N); 38.97 (CH_2_ph).

MS (MALDI, positive mode, matrix DHB) m/z: 386.46 (M + Na)^+^. Elemental analysis: calculated for C_21_H_21_N_3_O_3_ (363.42): % C, 69.41; % H, 5.82; % N, 11.56. Found: % C, 69.46; % H, 5.88; % N, 11.59.

##### General procedure for preparation of methyl-3-[2-(1,4-dioxo-3-phenyl-3,4-dihydro-1*H*-phthalazine-2-yl)-acetyl amino] alkanoate 7a–c

A cold solution at (− 5 °C) of acetohydrazide **4** (3.08 g, 10 mmol) in acetic acid (60 mL) and hydrochloric acid (5N, 30 mL) was added portion wise under stirring to a cold solution (0 °C) of sodium nitrite (0.7 g, 10 mmol) in water (30 mL). After stirring at the same temperature for 30 min, the in situ generated azide was extracted with cold ethyl acetate and washed successively with cold water and 5% Na_2_CO_3_.

After drying over anhydrous sodium sulphate, the azide was used without further purification in the next step. Amino acids methyl ester hydrochloride (15 mmol); glycine, methionine and valine which were placed with triethyl amine (1 g, 10 mmol) in ethyl acetate solution at (− 5 °C) for 20 min. Then the amino acid methyl ester hydrochloride solution was added to the previously prepared cold dried solution of the azide. Afterwards, the mixture was kept 12 h in the refrigerator and then at room temperature for another 12 h. The reaction mixture was filtered and the filtrated solution washed with 0.1N HCl, 5% Na_2_CO_3_ and water then dried over anhydrous sodium sulphate, the solvent was evaporated in vacuum and the residue was crystallized from ethyl acetate-petroleum ether to give products **7a–c**.

##### Synthesis of methyl (2-(4-benzyl-1-oxophthalazin-2(1*H*)-yl) acetyl) glycinate (7a)

White crystals (85%), m.p. 166–168 °C, ^1^H-NMR (400 MHz, CDCl_3_), (δ, ppm), (*J*, Hz): 8.35–8.38 (m, 1H, ArH); 7.60–7.68 (m, 3H, ArH); 7.18–7.21 (m, 4H, ArH); 7.11–7.15 (m, 1H, ArH); 6.71 (brs, 1H, NH); 4.90 (s, 2H, CH_2_CO); 4.23 (s, 2H, CH_2_ph); 4.00–4.01 (d, *J* = 5.2, 2H, CH_2_NH); 3.64 (s, 3H, OCH_3_).^13^C-NMR: 169.99 (C=O); 167.72 (C=O); 159.78 (C=O); 146.45 (C-Ar); 137.55 (C-Ar); 133.28 (CH-Ar); 131.50 (CH-Ar); 129.44 (C-Ar); 128.75 (2 CH-Ar); 128.44 (2 CH-Ar); 128.03 (C-Ar); 127.37 (CH-Ar);126.78 (CH-Ar); 125.46 (CH-Ar); 54.80 (CH_2_CO); 52.27 (OCH_3_); 41.29 (CH_2_NH); 38.91 (CH_2_ph).

MS (MALDI, positive mode, matrix DHB) m/z: 388.41 (M + Na)^+^. Elemental analysis: calculated for C_20_H_19_N_3_O_4_ (365.39): % C, 65.74; % H, 5.24; % N, 11.50. Found: % C, 65.66; % H, 5.18; % N, 11.43.

##### Synthesis of methyl (2-(4-benzyl-1-oxophthalazin-2(1*H*)-yl) acetyl) methioninate (7b)

White crystals (81%), m.p. 232–234 °C, ^1^H-NMR (400 MHz, CDCl_3_), (δ, ppm), (*J*, Hz): 8.37–8.39 (m, 1H, ArH); 7.62–7.68 (m, 3H, ArH); 7.19–7.22 (m, 4H, ArH); 7.12–7.15 (m, 1H, ArH); 6.80–6.82 (d, *J* = 7.2, 1H, NH); 4.84–4.94 (m, 2H, CH_2_CO); 4.66–4.71 (q, 1H, CHNH); 4.24 (s, 2H, CH_2_ph); 3.64 (s, 3H, OCH_3_); 2.43 (m, 2H, CH_2_S); 2.08–2.13 (m, 1H, CH_2_CH_2_S); 1.88–1.97 (m, 1H, CH_2_CH_2_S); 1.55 (s, 3H, SCH_3_). ^13^C-NMR: 172.00 (C=O); 167.34 (C=O); 159.72 (C=O); 146.45 (C-Ar); 137.53 (C-Ar); 133.28 (CH-Ar); 131.51 (CH-Ar); 129.44 (C-Ar); 128.76 (2 CH-Ar); 128.40 (2 CH-Ar); 128.07 (C-Ar); 127.39 (CH-Ar);126.79 (CH-Ar); 125.49 (CH-Ar); 54.90 (CH_2_CO); 52.46 (OCH_3_); 51.75 (CHNH); 38.94 (CH_2_ph); 31.53 (CH_2_CH_2_S); 29.90 (CH_2_S); 15.35 (SCH_3_).

MS (MALDI, positive mode, matrix DHB) m/z: 462.56 (M + Na)^+^. Elemental analysis: calculated for C_23_H_25_N_3_O_4_S (439.53): % C, 62.85; % H, 5.73; % N, 9.56; % S, 7.29. Found: % C, 62.81; % H, 5.70; % N, 9.52; % S, 7.23.

##### Synthesis of methyl (2-(4-benzyl-1-oxophthalazin-2(1*H*)-yl) acetyl) valinate (7c)

White crystals (86%), m.p. 150–152 °C, 1H-NMR (400 MHz, CDCl3), (δ, ppm), (*J*, Hz): 8.47–8.49 (m, 1H, ArH); 7.70–7.75 (m, 3H, ArH); 7.27–7.30 (m, 4H, ArH); 7.20–7.25 (m, 1H, ArH); 6.71–6.74 (d, *J* = 8.1, 1H, NH); 4.98 (s, 2H, CH_2_CO); 4.59–4.63 (dd, *J* = 6.4, *J* = 6.4, 1H, CHNH); 4.32 (s, 2H, CH_2_ph); 3.70 (s, 3H, OCH3); 2.16–2.21 (m, 1H, CH_3_CHCH_3_); 0.87–0.94 (d, *J* = 6.9, 6H, 2 CH_3_CH). ^13^C-NMR: 172.12 (C=O); 167.45 (C=O); 159.79 (C=O); 146.42 (C-Ar); 137.56 (C-Ar); 133.33 (CH-Ar); 131.55 (CH-Ar); 129.40 (C-Ar); 128.77 (2 CH-Ar); 128.39 (2 CH-Ar); 128.02 (C-Ar); 127.41 (CH-Ar); 126.80 (CH-Ar); 125.54 (CH-Ar); 57.20 (CHNH); 55.00 (CH_2_CO); 52.15 (OCH_3_); 38.97 (CH_2_ph); 31.38 (CH_3_CHCH_3_); 18.90 (CH_3_CH); 17.74 (CH_3_CH).

MS (MALDI, positive mode, matrix DHB) m/z: 430.49 (M + Na)^+^. Elemental analysis: calculated for C_23_H_25_N_3_O_4_ (407.47): % C, 67.80; % H, 6.18; % N, 10.31. Found: % C, 67.86; % H, 6.26; % N, 10.34.

##### Synthesis of hydrazide 8a

To a solution of ester **7a** (3.65 g, 0.01 mol) in ethyl alcohol (30 mL) was added hydrazine hydrate (1.6 mL, 0.05 mol). The reaction mixture was refluxed for 6 h, cooled and the white precipitate filtered and recrystallized from ethanol to obtain the corresponding hydrazide 2-(4-benzyl-1-oxophthalazin-2(1*H*)-yl)-*N*-(2-hydrazineyl-2-oxoethyl) acetamide **8a**.

##### Synthesis of 2-(4-benzyl-1-oxophthalazin-2(1*H*)-yl)-*N*-(2-hydrazineyl-2-oxo ethyl) acetamide (8a)

White crystals (84%), m.p. 202–204 °C, ^1^H-NMR (400 MHz, DMSO), (δ, ppm), (*J*, Hz): 9.02 (brs, 1H, NH); 8.38–8.41 (m, 1H, ArH); 8.27–8.29 (m, 1H, ArH); 7.91–7.93 (brs, 1H, NH); 7.80–7.88 (m, 2H, ArH); 7.34–7.36 (m, 2H, ArH); 7.27–7.31 (m, 2H, ArH); 7.18–7.21 (m, 1H, ArH); 4.87 (s, 2H, CH_2_CO); 4.32 (s, 2H, CH_2_ph); 4.23 (d, *J* = 5.2, 2H, CH_2_NH); 3.74–3.75 (d, *J* = 5.6, 2H, NH_2_). ^13^C-NMR: 168.38 (C=O); 167.73 (C=O); 159.08 (C=O); 145.48 (C-Ar); 138.56 (C-Ar); 133.82 (CH-Ar); 132.14 (CH-Ar); 129.38 (C-Ar); 129.01 (2 CH-Ar); 128.84 (2 CH-Ar); 128.08 (C-Ar); 126.95 (CH-Ar);126.85 (CH-Ar); 126.27 (CH-Ar); 53.98 (CH_2_CO); 41.43 (CH_2_NH); 38.13 (CH_2_ph).

MS (MALDI, positive mode, matrix DHB) m/z: 388.42 (M + Na)^+^. Elemental analysis: calculated for C_19_H_19_N_5_O_3_ (365.39): % C, 62.46; % H, 5.24; % N, 19.17. Found: % C, 62.44; % H, 5.21; % N, 19.12.

##### General procedure for synthesis of 2-(4-benzyl-1-oxophthalazin-2(1*H*)-yl)-*N*-(2-oxo-2-(alkyl amino) ethyl) acetamide 10a–h

Under azide coupling method as previewed before, A cold solution at (− 5 °C) of 2-(4-benzyl-1-oxophthalazin-2(1*H*)-yl)-N-(2-hydrazineyl-2-oxoethyl) acetamide **(8a)** (3.65 g, 10 mmol) in acetic acid (60 mL) and hydrochloric acid (5N, 30 mL) was added portion wise under stirring to a cold solution (0 °C) of sodium nitrite (0.7 g, 0.01 mol) in water (30 mL). After stirring at the same temperature for 30 min, the in situ generated azide was extracted with cold ethyl acetate and washed successively with cold water and 5% Na_2_CO_3_.

After drying over anhydrous sodium sulphate, the azide was used without further purification in the next step. Amines (12 mmol) were added to the previously prepared cold dried solution of the azide. Afterwards, the mixture was kept 12 h in the refrigerator and then at room temperature for another 12 h. The reaction mixture was filtered and the filtrated solution washed with 0.1N HCl, 5% Na_2_CO_3_ and water then dried over anhydrous sodium sulphate, the solvent was evaporated in vacuum and the residue was crystallized from ethyl acetate-petroleum ether to give products **10a–h**.

##### Synthesis of *N*-benzyl-2-(2-(4-benzyl-1-oxophthalazin-2(1*H*)-yl)acetamido) acetamide (10a)

Off-white crystals (83%), m.p. 186–188 °C, ^1^H-NMR (400 MHz, CDCl_3_), (δ, ppm), (*J*, Hz): 8.23–8.25 (m, 1H, ArH); 7.68–7.76 (m, 3H, ArH); 7.27–7.33 (m, 9H, ArH); 7.22–7.25 (m, 1H, ArH); 7.04 (brs, 2H, 2 NH); 4.92 (s, 2H, NCH_2_CO); 4.43–4.45 (d, *J* = 5.6, 2H, NHCH_2_ph); 4.31 (s, 2H, CH_2_ph); 4.01–4.03 (d, *J* = 5.6, 2H, NHCH_2_CO). ^13^C-NMR: 168.65 (C=O); 168.02 (C=O); 159.93 (C=O); 146.66 (C-Ar); 138.00 (C-Ar); 137.44 (C-Ar); 133.37 (CH-Ar); 131.51 (CH-Ar); 129.52 (C-Ar); 128.78 (2 CH-Ar); 128.57 (2 CH-Ar); 128.41 (2 CH-Ar); 127.83 (C-Ar); 127.72 (2 CH-Ar); 127.32 (CH-Ar); 127.19 (CH-Ar);126.84 (CH-Ar); 125.47 (CH-Ar); 55.76 (NCH_2_CO); 43.51 (NHCH_2_CO); 43.35 (NHCH_2_ph); 38.88 (CH_2_ph).

MS (MALDI, positive mode, matrix DHB) m/z: 463.52 (M + Na)^+^. Elemental analysis: calculated for C_26_H_24_N_4_O_3_ (440.50): % C, 70.89; % H, 5.49; % N, 12.72. Found: % C, 70.93; % H, 5.56; % N, 12.77.

##### Synthesis of 2-(4-benzyl-1-oxophthalazin-2(1*H*)-yl)-*N*-(2-oxo-2-(propyl amino) ethyl) acetamide (10b)

White crystals (86%), m.p. 152–154 °C, ^1^H-NMR (400 MHz, CDCl_3_), (δ, ppm), (*J*, Hz): 8.41–8.44 (m, 1H, ArH); 7.72–7.79 (m, 3H, ArH); 7.28–7.31 (m, 4H, ArH); 7.22–7.25 (m, 1H, ArH); 6.88 (brs, 1H, NH); 6.62 (brs, 1H, NH); 4.94 (s, 2H, NCH_2_CO); 4.34 (s, 2H, CH_2_ph); 3.98–3.99 (d, *J* = 4.8, 2H, NHCH_2_CO); 3.19–3.24 (q, 2H, NHCH_2_CH_2_); 1.52–1.57 (sextet, 2H, CH_2_CH_2_CH_3_); 0.89–0.92 (t, *J* = 7.2, 3H, CH_3_). ^13^C-NMR: 168.46 (C=O); 167.89 (C=O); 159.93 (C=O); 146.62 (C-Ar); 137.44 (C-Ar); 133.41 (CH-Ar); 131.57 (CH-Ar); 129.57 (C-Ar); 128.78 (2 CH-Ar); 128.41 (2 CH-Ar); 127.94 (C-Ar); 127.17 (CH-Ar);126.84 (CH-Ar); 125.55 (CH-Ar); 55.71 (NCH_2_CO); 43.42 (NHCH_2_CO); 41.37 (NHCH_2_CH_2_); 38.91 (CH_2_ph); 22.57 (CH_2_CH_2_CH_3_);11.31 (CH_3_).

MS (MALDI, positive mode, matrix DHB) m/z: 415.47 (M + Na)^+^. Elemental analysis: calculated for C_22_H_24_N_4_O_3_ (392.46): % C, 67.33; % H, 6.16; % N, 14.28. Found: % C, 67.36; % H, 6.22; % N, 14.32.

##### Synthesis of 2-(4-benzyl-1-oxophthalazin-2(1*H*)-yl)-*N*-(2-(butyl amino)-2-oxoethyl) acetamide (10c)

Off-white crystals (85%), m.p. 156–158 °C, ^1^H-NMR (400 MHz, CDCl_3_), (δ, ppm), (*J*, Hz): 8.43–8.45 (m, 1H, ArH); 7.73–7.80 (m, 3H, ArH); 7.29–7.32 (m, 4H, ArH); 7.23–7.27 (m, 1H, ArH); 6.76 (brs, 1H, NH); 6.50 (brs, 1H, NH); 4.94 (s, 2H, NCH_2_CO); 4.34 (s, 2H, CH_2_ph); 3.99 (d, *J* = 5.2, 2H, NHCH_2_CO); 3.24–3.29 (q, 2H, NHCH_2_CH_2_); 1.48–1.54 (qn, 2H, CH_2_CH_2_CH_2_); 1.32–1.37 (sextet, 2H, CH_2_CH_2_CH_3_); 0.90–0.93 (t, *J* = 7.2, 3H, CH_3_). ^13^C-NMR: 168.34 (C=O); 167.85 (C=O); 159.94 (C=O); 146.67 (C-Ar); 137.42 (C-Ar); 133.44 (CH-Ar); 131.59 (CH-Ar); 129.58 (C-Ar); 128.79 (2 CH-Ar); 128.42 (2 CH-Ar); 127.93 (C-Ar); 127.21 (CH-Ar);126.86 (CH-Ar); 125.56 (CH-Ar); 55.80 (NCH_2_CO); 43.43 (NHCH_2_CO); 39.41 (NHCH_2_CH_2_); 38.92 (CH_2_ph); 31.41 (CH_2_CH_2_CH_2_); 20.01 (CH_2_CH_2_CH_3_);13.67 (CH_3_).

MS (MALDI, positive mode, matrix DHB) m/z: 429.53 (M + Na)^+^. Elemental analysis: calculated for C_23_H_26_N_4_O_3_ (406.49): % C, 67.96; % H, 6.45; % N, 13.78. Found: % C, 67.99; % H, 6.55; % N, 13.86.

##### Synthesis of 2-(4-benzyl-1-oxophthalazin-2(1*H*)-yl)-*N*-(2-(cyclohexylamino)-2-oxoethyl) acetamide (10d)

White crystals (87%), m.p. 157–158 °C, ^1^H-NMR (400 MHz, CDCl_3_), (δ, ppm), (*J*, Hz): 8.43–8.45 (m, 1H, ArH); 7.73–7.79 (m, 3H, ArH); 7.28–7.32 (m, 4H, ArH); 7.22–7.25 (m, 1H, ArH); 6.81 (brs, 1H, NH); 6.37–6.38 (d, *J* = 7.2, 1H, NH); 4.94 (s, 2H, NCH_2_CO); 4.34 (s, 2H, CH_2_ph); 3.97–3.98 (d, *J* = 5.2, 2H, NHCH_2_CO); 3.75–3.76 (sextet, 1H, NHCHCH_2_); 1.86–1.89 (m, 2H, CH_2_); 1.69–1.72 (m, 2H, CH_2_); 1.60–1.63 (m, 1H, CH); 1.25–1.36 (m, 2H, CH_2_); 1.15–1.22 (m, 3H, CH_2_ / CH). ^13^C-NMR: 167.75 (C=O); 167.40 (C=O); 159.87 (C=O); 146.59 (C-Ar); 137.44 (C-Ar); 133.40 (CH-Ar); 131.56 (CH-Ar); 129.59 (C-Ar); 128.78 (2 CH-Ar); 128.42 (2 CH-Ar); 127.95 (C-Ar); 127.21 (CH-Ar); 126.84 (CH-Ar); 125.55 (CH-Ar); 55.73 (NCH_2_CO); 48.44 (NHCH_2_CO); 43.45 (NHCHCH_2_); 38.93 (CH_2_ph); 32.83 (2 CH_2_);25.53 (CH_2_); 24.81 (2 CH_2_).

MS (MALDI, positive mode, matrix DHB) m/z: 455.54 (M + Na)^+^. Elemental analysis: calculated for C_25_H_28_N_4_O_3_ (432.52): % C, 69.42; % H, 6.53; % N, 12.95. Found: % C, 69.48; % H, 6.61; % N, 12.94.

##### Synthesis of 2-(4-benzyl-1-oxophthalazin-2(1*H*)-yl)-*N*-(2-oxo-2-(tetradecyl amino) ethyl) acetamide (10e)

Off-white crystals (82%), m.p. 149–150 °C, ^1^H-NMR (400 MHz, CDCl_3_), (δ, ppm), (*J*, Hz): 8.40–8.42 (m, 1H, ArH); 7.71–7.77 (m, 3H, ArH); 7.29–7.30 (m, 4H, ArH); 7.21–7.24 (m, 1H, ArH); 6.70 (brs, 1H, NH); 6.62 (brs, 1H, NH); 4.94 (s, 2H, NCH_2_CO); 4.33 (s, 2H, CH_2_ph); 3.96–3.97 (d, *J* = 3.2, 2H, NHCH_2_CO); 3.20–3.25 (q, 2H, NHCH_2_CH_2_); 2.70–2.74 (t, *J* = 6.8, 1H, NH); 2.35 (m, 2H, CH_2_); 1.48 (sextet, 2H, CH_2_CH_2_CH_3_); 1.28 (m, 20H, 10 CH_2_); 0.88–0.91 (t, *J* = 6.8, 3H, CH_3_). ^13^C-NMR: 168.64 (C=O); 167.96 (C=O); 159.88 (C=O); 146.49 (C-Ar); 137.47 (C-Ar); 133.33 (CH-Ar); 131.47 (CH-Ar); 129.57 (C-Ar); 128.76 (2 CH-Ar); 128.39 (2 CH-Ar); 127.93 (C-Ar); 127.14 (CH-Ar); 126.81 (CH-Ar); 125.52 (CH-Ar); 55.64 (NCH_2_CO); 43.34 (NHCH_2_CO); 42.06 (CH_2_); 39.74 (NHCH_2_CH_2_); 38.91 (CH_2_ph); 33.38 (CH_2_); 31.89 (2 CH_2_); 29.31–29.63 (4 CH_2_); 26.85 (2 CH_2_); 22.64 (2 CH_2_); 14.04 (CH_3_).

MS (MALDI, positive mode, matrix DHB) m/z: 569.80 (M + Na)^+^. Elemental analysis: calculated for C_33_H_46_N_4_O_3_ (546.76): % C, 72.49; % H, 8.48; % N, 10.25. Found: % C, 72.53; % H, 8.45; % N, 10.36.

##### Synthesis of *N*-allyl-2-(2-(4-benzyl-1-oxophthalazin-2(1*H*)-yl) acetamido) acetamide (10f)

White crystals (88%), m.p. 188–190 °C, ^1^H-NMR (400 MHz, CDCl_3_), (δ, ppm), (*J*, Hz): 8.42–8.44 (m, 1H, ArH); 7.73–7.77 (m, 3H, ArH); 7.28–7.32 (m, 4H, ArH); 7.24–7.25 (m, 1H, ArH); 6.83 (brs, 1H, NH); 6.63 (brs, 1H, NH); 5.81–5.88 (m, 1H, CH=CH_2_); 5.11–5.22 (dd, *J* = 17.2, *J* = 10.4, 2H, CH_2_=CH); 4.95 (s, 2H, NCH_2_CO); 4.34 (s, 2H, CH_2_ph); 4.02 (d, *J* = 5.2, 2H, NHCH_2_CO); 3.90 (t, *J* = 5.6, 2H, NHCH_2_CH). ^13^C-NMR: 168.39 (C=O); 167.98 (C=O); 159.97 (C=O); 146.73 (C-Ar); 137.41 (C-Ar); 133.77 (CH = CH_2_); 133.46 (CH-Ar); 131.60 (CH-Ar); 129.57 (C-Ar); 128.79 (2 CH-Ar); 128.41 (2 CH-Ar); 127.90 (C-Ar); 127.26 (CH-Ar);126.86 (CH-Ar); 125.54 (CH-Ar); 116.50 (CH_2_=CH); 55.80 (NCH_2_CO); 43.43 (NHCH_2_CO); 41.98 (NHCH_2_CH); 38.92 (CH_2_ph).

MS (MALDI, positive mode, matrix DHB) m/z: 413.47 (M + Na)^+^. Elemental analysis: calculated for C_22_H_22_N_4_O_3_ (390.44): % C, 67.68; % H, 5.68; % N, 14.35. Found: % C, 67.66; % H, 5.61; % N, 14.23.

##### Synthesis of 2-(4-benzyl-1-oxophthalazin-2(1*H*)-yl)-*N*-(2-oxo-2-(piperidin-1-yl) ethyl) acetamide (10g)

White crystals (79%), m.p. 138–140 °C, ^1^H-NMR (400 MHz, CDCl_3_), (δ, ppm), (*J*, Hz): 8.44–8.46 (m, 1H, ArH); 7.70–7.71 (m, 3H, ArH); 7.30–7.32 (m, 4H, ArH); 7.22–7.23 (m, 1H, ArH); 7.07 (brs, 1H, NH); 5.00 (s, 2H, NCH_2_CO); 4.34 (s, 2H, CH_2_ph); 4.10–4.11 (d, *J* = 5.2, 2H, NHCH_2_CO); 3.56 (m, 2H, CH_2_N); 3.40 (m, 2H, CH_2_N); 1.56–1.65 (m, 6H, 3 CH_2_). ^13^C-NMR: 168.34 (C=O); 167.46 (C=O); 159.89 (C=O); 146.43 (C-Ar); 137.56 (C-Ar); 133.34 (CH-Ar); 131.56 (CH-Ar); 129.57 (C-Ar); 128.76 (2 CH-Ar); 128.42 (2 CH-Ar); 127.92 (C-Ar); 127.21 (CH-Ar);126.67 (CH-Ar); 125.53 (CH-Ar); 55.74 (NCH_2_CO);45.86 (CH_2_N); 43.64 (CH_2_N); 43.34 (NHCH_2_CO); 39.08 (CH_2_ph); 26.55 (CH_2_CH_2_N);25.41 (CH_2_CH_2_N); 23.94 (CH_2_CH_2_CH_2_N).

MS (MALDI, positive mode, matrix DHB) m/z: 441.54 (M + Na)^+^. Elemental analysis: calculated for C_24_H_26_N_4_O_3_ (418.50): % C, 68.88; % H, 6.26; % N, 13.39. Found: % C, 68.92; % H, 6.31; % N, 13.48.

##### Synthesis of 2-(4-benzyl-1-oxophthalazin-2(1*H*)-yl)-*N*-(2-morpholino-2-oxo ethyl) acetamide (10h)

White crystals (81%), m.p. 148–150 °C, ^1^H-NMR (400 MHz, CDCl_3_), (δ, ppm), (*J*, Hz): 8.47–8.48 (m, 1H, ArH); 7.72–7.73 (m, 3H, ArH); 7.28–7.32 (m, 4H, ArH); 7.23–7.24 (m, 1H, ArH); 7.10 (brs, 1H, NH); 5.01 (s, 2H, NCH_2_CO); 4.34 (s, 2H, CH_2_ph); 4.13 (d, *J* = 5.2, 2H, NHCH_2_CO); 3.64–3.69 (m, 6H, 2 CH_2_O /CH_2_N); 3.43 (m, 2H, CH_2_N). ^13^C-NMR: 167.44 (C=O); 166.34 (C=O); 159.67 (C=O); 146.34 (C-Ar); 137.62 (C-Ar); 133.20 (CH-Ar); 131.47 (CH-Ar); 129.49 (C-Ar); 128.74 (2 CH-Ar); 128.44 (2 CH-Ar); 128.11 (C-Ar); 127.46 (CH-Ar);126.74 (CH-Ar); 125.50 (CH-Ar); 66.67 (CH_2_O); 66.33 (CH_2_O); 54.60 (NCH_2_CO);44.86 (NHCH_2_CO); 42.36 (CH_2_N); 41.25 (CH_2_N); 39.01 (CH_2_ph).

MS (MALDI, positive mode, matrix DHB) m/z: 443.50 (M + Na)^+^. Elemental analysis: calculated for C_23_H_24_N_4_O_4_ (420.47): % C, 65.70; % H, 5.75; % N, 13.33. Found: % C, 65.64; % H, 5.65; % N, 13.26.

##### General procedure for preparation of methyl (2-(4-benzyl-1-oxophthalazin-2(1*H*)-yl) acetyl) glycyl alkanoate 11a–d

A cold solution at (− 5 °C) of 2-(4-benzyl-1-oxophthalazin-2(1*H*)-yl)-N-(2-hydrazineyl-2-oxoethyl) acetamide **(8a)** (3.65 g, 10 mmol) in acetic acid (60 mL) and hydrochloric acid (5N, 30 mL) was added portion wise under stirring to a cold solution (0 °C) of sodium nitrite (0.7 g, 10 mmol) in water (30 mL). After stirring at the same temperature for 30 min, the in situ generated azide was extracted with cold ethyl acetate and washed successively with cold water and 5% Na_2_CO_3_.

After drying over anhydrous sodium sulphate, the azide was used without further purification in the next step. Amino acids methyl ester hydrochloride (15 mmol); glycine, β-alanine, methionine and valine which were placed with triethyl amine (1 g, 10 mmol) in ethyl acetate solution at (− 5 °C) for 20 min. Then the amino acid methyl ester hydrochloride solution was added to the previously prepared cold dried solution of the azide. Afterwards, the mixture was kept 12 h in the refrigerator and then at room temperature for another 12 h. The reaction mixture was filtered, and the filtrated solution washed with 0.1N HCl, 5% Na_2_CO_3_ and water then dried over anhydrous sodium sulphate, the solvent was evaporated in vacuum and the residue was crystallized from ethyl acetate-petroleum ether to give products **11a–d**.

##### Synthesis of methyl (2-(4-benzyl-1-oxophthalazin-2(1*H*)-yl) acetyl) glycyl glycinate (11a)

White crystals (88%), m.p. 162–164 °C, ^1^H-NMR (400 MHz, CDCl_3_), (δ, ppm), (*J*, Hz): 8.42 (m, 1H, ArH); 7.73–7.75 (m, 3H, ArH); 7.29–7.31 (m, 4H, ArH); 7.23–7.24 (m, 1H, ArH); 7.08 (brs, 2H, 2 NH); 4.97 (s, 2H, NCH_2_CO); 4.33 (s, 2H, CH_2_ph); 4.04–4.05 (d, *J* = 4.8, 4H, 2 NHCH_2_CO); 3.72 (s, 3H, OCH_3_).

^13^C-NMR: 172.11 (C=O); 168.08 (C=O); 167.63 (C=O); 159.94 (C=O); 146.70 (C-Ar); 137.46 (C-Ar); 133.39 (CH-Ar); 131.56 (CH-Ar); 129.55 (C-Ar); 128.78 (2 CH-Ar); 128.42 (2 CH-Ar); 127.93 (C-Ar); 127.26 (CH-Ar);126.83 (CH-Ar); 125.49 (CH-Ar); 55.63 (NCH_2_CO); 52.27 (OCH_3_); 43.13 (NHCH_2_COO); 41.17 (NHCH_2_CO); 38.89 (CH_2_ph).

MS (MALDI, positive mode, matrix DHB) m/z: 445.46 (M + Na)^+^. Elemental analysis: calculated for C_22_H_22_N_4_O_5_ (422.44): % C, 62.55; % H, 5.25; % N, 13.26. Found: % C, 62.49; % H, 5.22; % N, 13.30.

##### Synthesis of methyl 3-(2-(2-(4-benzyl-1-oxophthalazin-2(1*H*)-yl) acetamido acetamido) propanoate (11b)

White crystals (85%), m.p. 108–110 °C, ^1^H-NMR (400 MHz, CDCl_3_), (δ, ppm), (*J*, Hz): 8.44–8.46 (m, 1H, ArH); 7.72–7.78 (m, 3H, ArH); 7.28–7.31 (m, 4H, ArH); 7.22–7.25 (m, 1H, ArH); 6.88 (brs, 2H, 2 NH); 4.95 (s, 2H, NCH_2_CO); 4.34 (s, 2H, CH_2_ph); 3.97–3.98 (d, *J* = 4.4, 2H, NHCH_2_CO); 3.67 (s, 3H, OCH_3_); 3.54–3.55 (q, 2H, NHCH_2_CH_2_CO); 2.56–2.59 (t, *J* = 6, 2H, NHCH_2_CH_2_CO). ^13^C-NMR: 172.50 (C=O); 168.57 (C=O); 167.88 (C=O); 159.93 (C=O); 146.62 (C-Ar); 137.47 (C-Ar); 133.38 (CH-Ar); 131.58 (CH-Ar); 129.55 (C-Ar); 128.77 (2 CH-Ar); 128.42 (2 CH-Ar); 127.98 (C-Ar); 127.28 (CH-Ar); 126.83 (CH-Ar); 125.51 (CH-Ar); 55.59 (NCH_2_CO); 51.73 (OCH_3_);43.25 (NHCH_2_CO); 38.91 (CH_2_ph); 35.18 (NHCH_2_CH_2_CO); 33.71 (NHCH_2_CH_2_CO).

MS (MALDI, positive mode, matrix DHB) m/z: 459.48 (M + Na)^+^. Elemental analysis: calculated for C_23_H_24_N_4_O_5_ (436.47): % C, 63.29; % H, 5.54; % N, 12.84. Found: % C, 63.35; % H, 5.63; % N, 12.82.

##### Synthesis of methyl (2-(4-benzyl-1-oxophthalazin-2(1*H*)-yl) acetyl) glycyl methioninate (11c)

Off-white crystals (84%), m.p. 130–132 °C, ^1^H-NMR (400 MHz, CDCl_3_), (δ, ppm), (*J*, Hz): 8.48 (m, 1H, ArH); 7.73–7.74 (m, 3H, ArH); 7.55–7.56 (brs, 1H, NH); 7.28 (m, 4H, ArH); 7.19–7.21 (brs, 1H, NH); 7.14–7.17 (m, 1H, ArH); 4.99 (q, 1H, NHCHCO); 4.32 (s, 2H, NCH_2_CO); 4.23–4.26 (d, *J* = 6, 2H, NHCH_2_CO); 4.18 (s, 2H, CH_2_ph); 3.76 (s, 3H, OCH_3_); 3.03–3.07 (t, *J* = 7.2, 2H, CH_2_S); 2.25 (q, 2H, CH_2_CH_2_S); 1.71 (s, 3H, SCH_3_). ^13^C-NMR: 172.18 (C=O); 168.60 (C=O); 167.79 (C=O); 159.84 (C=O); 146.45 (C-Ar); 137.47 (C-Ar); 133.34 (CH-Ar); 131.54 (CH-Ar); 130.80 (C-Ar); 128.78 (2 CH-Ar); 128.42 (2 CH-Ar); 127.86 (C-Ar); 127.43 (CH-Ar);126.81 (CH-Ar); 125.53 (CH-Ar); 57.41 (NHCHCO); 55.56 (NCH_2_CO); 52.08 (OCH_3_);51.67 (NHCH_2_CO); 43.34 (CH_2_CH_2_S); 38.92 (CH_2_ph); 31.49 (CH_2_S); 16.85 (SCH_3_).

MS (MALDI, positive mode, matrix DHB) m/z: 519.61 (M + Na)^+^. Elemental analysis: calculated for C_25_H_28_N_4_O_5_S (496.58): % C, 60.47; % H, 5.68; % N, 11.28; % S, 6.46. Found: % C, 60.33; % H, 5.57; % N, 11.15; % S, 6.40.

##### Synthesis of methyl (2-(4-benzyl-1-oxophthalazin-2(1*H*)-yl) acetyl) glycyl valinate (11d)

White crystals (82%), m.p. 190–192 °C, ^1^H-NMR (400 MHz, CDCl_3_), (δ, ppm), (*J*, Hz): 8.45 (m, 1H, ArH); 7.72–7.76 (m, 3H, ArH); 7.28–7.32 (m, 4H, ArH); 7.22–7.24 (m, 1H, ArH); 6.88 (brs, 1H, NH); 6.65 (brs, 1H, NH); 4.98 (s, 2H, NCH_2_CO); 4.34 (s, 2H, CH_2_ph); 4.25 (t, *J* = 5.2, 1H, NHCHCO); 3.71 (d, *J* = 5.2, 2H, NHCH_2_CO); 3.51 (s, 3H, OCH_3_); 1.45 (m, 1H, CH_3_CHCH_3_); 0.92–0.95 (d, *J* = 6.4, 6H, 2 CH_3_CH). ^13^C-NMR: 172.48 (C=O); 168.52 (C=O); 167.82 (C=O); 159.91 (C=O); 146.44 (C-Ar); 137.36 (C-Ar); 133.38 (CH-Ar); 131.56 (CH-Ar); 130.82 (C-Ar); 128.79 (2 CH-Ar); 128.45 (2 CH-Ar); 127.87 (C-Ar); 127.40 (CH-Ar);126.84 (CH-Ar); 125.54 (CH-Ar); 57.43 (NHCHCO); 55.51 (NCH_2_CO); 52.08 (OCH_3_);43.30 (NHCH_2_CO); 38.94 (CH_2_ph); 29.68 (CH_3_CHCH_3_); 18.93 (CH_3_CH); 17.88 (CH_3_CH).

MS (MALDI, positive mode, matrix DHB) m/z: 487.56 (M + Na)^+^. Elemental analysis: calculated for C_25_H_28_N_4_O_5_ (464.52): % C, 64.64; % H, 6.08; % N, 12.06. Found: % C, 64.76; % H, 6.15; % N, 12.16.

##### Synthesis of 2-(4-benzyl-1-oxophthalazin-2(1*H*)-yl)-*N*-2-(3,5-diamino-1*H*-pyrazol-1-yl)-2-oxoethyl) acetamide (12a)

A mixture of hydrazide **8a** (3.65 g, 0.01 mol) and malononitrile (1.32 g, 0.02 mol) in ethanol (30 mL) was refluxed for 8 h. By cooling the solid product formed, filtered off and recrystallized from ethanol solvent gave compound **12a**.

White crystals (85%), m.p. 236–238 °C, ^1^H-NMR (400 MHz, DMSO), (δ, ppm), (*J*, Hz): 9.02 (brs, 1H, NHCH_2_CO); 8.27–8.28 (m, 1H, ArH); 7.90–7.92 (m, 1H, ArH); 7.81–7.85 (m, 2H, ArH); 7.31–7.32 (m, 3H, ArH); 7.18 (m, 2H, ArH); 7.10–7.12 (s, 1H, NH_2_-C=CH-C-NH_2_); 5.46–5.47 (d, *J* = 5.2, 2H, NHCH_2_CO); 4.86 (s, 2H, NCH_2_CO); 4.32 (brs, 2H, NH_2_); 4.26 (s, 2H, CH_2_ph); 3.52 (brs, 2H, NH_2_). ^13^C-NMR: 171.42 (C=O); 168.34 (C=O); 159.21 (C=O); 148.14 (C-NH_2_); 148.36 (C-NH_2_); 146.38 (C-Ar); 137.32 (C-Ar); 133.30 (CH-Ar); 131.64 (CH-Ar); 130.85 (C-Ar); 128.78 (2 CH-Ar); 128.43 (2 CH-Ar); 128.09 (C-Ar); 127.49 (CH-Ar);126.76 (CH-Ar); 125.34 (CH-Ar); 110.35 (NH_2_-C=CH-C-NH_2_); 55.28 (NCH_2_CO); 42.82 (NHCH_2_CO); 38.64 (CH_2_ph).

MS (MALDI, positive mode, matrix DHB) m/z: 454.49 (M + Na)^+^. Elemental analysis: calculated for C_22_H_21_N_7_O_3_ (431.46): % C, 61.24; % H, 4.91; % N, 22.73. Found: % C, 61.32; % H, 5.02; % N, 22.80.

##### Synthesis of 2-(4-benzyl-1-oxophthalazin-2(1*H*)-yl)-*N*-(2-(2-cyclohexylidene hydrazineyl)-2-oxoethyl) acetamide (12b)

A mixture of hydrazide molecule **8a** (3.65 g, 0.01 mol) and cyclohexanone (1.96 g, 0.02 mol) in ethanol (30 mL) was refluxed for 8 h. By cooling the solid product formed, filtered off and recrystallized from ethanol solvent gave compound **12b**.

White crystals (89%), m.p. 233–234 °C, ^1^H-NMR (400 MHz, DMSO), (δ, ppm), (*J*, Hz): 9.01 (brs, 1H, CONHNH); 8.40 (m, 1H, ArH); 8.26–8.28 (m, 1H, ArH); 7.90–7.91 (m, 1H, ArH); 7.78–7.86 (m, 2H, ArH); 7.30–7.34 (m, 3H, ArH); 7.16–7.20 (m, 1H, ArH); 7.16–7.20 (t, *J* = 5.2, 1H, NHCH_2_CO); 7.08–7.12 (m, 1H, NH-C=CH-CH_2_); 5.46–5.47 (d, *J* = 5.2, 2H, NHCH_2_CO); 4.85 (s, 2H, NCH_2_CO); 4.32 (brs, 1H, NHNH-C=CH-CH_2_); 4.25 (s, 2H, CH_2_ph); 2.37 (t, *J* = 6, 2H, NH-C-CH_2_); 1.65 (q, 2H, NH-C=CH-CH_2_); 1.14 (qn, 4H, 2 CH_2_). ^13^C-NMR: 170.62 (C=O); 168.32 (C=O); 159.18 (C=O); 150.11 (NH-C=CH-CH_2_); 145.83 (C-Ar); 138.34 (C-Ar); 133.77 (CH-Ar); 131.93 (CH-Ar); 129.52 (C-Ar); 129.06 (2 CH-Ar); 128.78 (2 CH-Ar); 128.07 (C-Ar); 127.04 (CH-Ar);126.87 (CH-Ar); 125.96 (CH-Ar); 102.45 (NH-C=CH-CH_2_); 54.86 (NCH_2_CO); 41.03 (NHCH_2_CO); 38.25 (CH_2_ph); 35.19 (NH-C-CH_2_); 34.32 (NH-C=CH-CH_2_); 26.50 (CH_2_); 22.71 (CH_2_).

MS (MALDI, positive mode, matrix DHB) m/z: 468.55 (M + Na)^+^. Elemental analysis: calculated for C_25_H_27_N_5_O_3_ (445.52): % C, 67.40; % H, 6.11; % N, 15.72. Found: % C, 67.33; % H, 6.01; % N, 15.63.

##### Synthesis of (E)-2-(4-benzyl-1-oxophthalazin-2(1*H*)-yl)-*N*-(2-(2-(1-(furan-2-yl) ethylidene) hydrazineyl)-2-oxoethyl) acetamide (12c)

A mixture of hydrazide molecule **8a** (3.65 g, 0.01 mol) and 2-furyl methyl ketone (2.2 g, 0.02 mol) in ethanol (30 mL) was refluxed for 8 h. By cooling the solid product formed, filtered off and recrystallized from ethanol solvent gave compound **12c**.

Faint brown crystals (91%), m.p. 209–210 °C, ^1^H-NMR (400 MHz, DMSO), (δ, ppm), (*J*, Hz): 10.64 (m, 1H, CH=CH-O); 8.50 (m, 1H, ArH); 8.27–8.29 (m, 1H, ArH); 7.91–7.93 (m, 1H, ArH); 7.81–7.88 (m, 2H, ArH); 7.75 (brs, 1H, CONHN=C); 7.35–7.37 (m, 2H, ArH); 7.27–7.31 (m, 2H, ArH); 7.17–7.21 (t, *J* = 5.2, 1H, NHCH_2_CO); 6.89 (m, 1H, CH=C-O); 6.58 (m, 1H, CH-CH=CH-O); 4.89 (s, 2H, NCH_2_CO); 4.32 (d, *J* = 5.2, 2H, NHCH_2_CO); 4.32 (s, 2H, CH_2_ph); 2.18 (s, 3H, CH_3_). ^13^C-NMR: 172.73 (C=O); 168.82 (C=O); 159.30 (C=O); 152.64 (N=C-CH_3_); 146.11 (C-Ar); 144.58 (CH=C-O); 144.18 (CH=CH-O); 140.32 (C-Ar); 138.39 (CH-Ar); 133.81 (CH-Ar); 131.92 (CH-Ar); 129.75 (C-Ar); 128.98 (2 CH-Ar); 128.83 (2 CH-Ar); 128.07 (C-Ar); 127.12 (CH-Ar);126.40 (CH-Ar); 112.19 (CH=CH-O); 110.85 (CH=C-O); 55.16 (NCH_2_CO); 42.88 (NHCH_2_CO); 38.54 (CH_2_ph); 13.32 (CH_3_).

MS (MALDI, positive mode, matrix DHB) m/z: 480.50 (M + Na)^+^. Elemental analysis: calculated for C_25_H_23_N_5_O_4_ (457.49): % C, 65.64; % H, 5.07; % N, 15.31. Found: % C, 65.67; % H, 5.11; % N, 15.25.

##### Synthesis of *N*-(2-(5-amino-3-oxo-2,3-dihydro-1*H*-pyrazol-1-yl)-2-oxoethyl)-2-(4-benzyl-1-oxophthalazin-2(1*H*)-yl) acetamide (12d)

A mixture of hydrazide molecule **8a** (3.65 g, 0.01 mol) and ethyl cyano acetate (2.26 g, 0.02 mol) in ethanol (30 mL) was refluxed for 8 h. By cooling the solid product formed, filtered off and recrystallized from ethanol solvent gave compound **12d**.

White crystals (84%), m.p. 237–238 °C, ^1^H-NMR (400 MHz, DMSO), (δ, ppm), (*J*, Hz): 9.02 (brs, 1H, N–NH-CO); 8.39 (m, 1H, ArH); 8.26–8.28 (m, 1H, ArH); 7.90–7.92 (m, 1H, ArH); 7.78–7.86 (m, 2H, ArH); 7.30–7.34 (m, 3H, ArH); 7.17–7.20 (m, 1H, ArH); 7.17–7.20 (t, *J* = 5.2, 1H, NHCH_2_CO); 7.08–7.12 (s, 1H, NH_2_-C=CH-CO); 5.46–5.47 (d, *J* = 5.2, 2H, NHCH_2_CO); 4.86 (s, 2H, NCH_2_CO); 4.32 (s, 2H, NH_2_-C=CH-CO); 4.25 (s, 2H, CH_2_ph). ^13^C-NMR: 171.78 (C=O); 167.36 (C=O); 166.29 (C=O); 158.89 (C=O); 151.93 (NH_2_-C=CH-CO); 145.33 (C-Ar); 138.45 (C-Ar); 133.97 (CH-Ar); 132.18 (CH-Ar); 129.18 (C-Ar); 128.91 (2 CH-Ar); 128.76 (2 CH-Ar); 128.09 (C-Ar); 126.88 (CH-Ar);126.21 (CH-Ar); 125.32 (CH-Ar); 112.19 (NH_2_-C=CH-CO); 56.34 (NCH_2_CO); 43.24 (NHCH_2_CO); 38.11 (CH_2_ph).

MS (MALDI, positive mode, matrix DHB) m/z: 455.48 (M + Na)^+^. Elemental analysis: calculated for C_22_H_20_N_6_O_4_ (432.44): % C, 61.10; % H, 4.66; % N, 19.43. Found: % C, 61.04; % H, 4.61; % N, 19.50.

##### Synthesis of 2-(4-benzyl-1-oxophthalazin-2(1*H*)-yl)-*N*-(2-(3,5-dimethyl-1*H*-pyrazol-1-yl)-2-oxoethyl) acetamide (12e)

A mixture of hydrazide molecule **8a** (3.65 g, 0.01 mol) and acetyl acetone (2 g, 0.02 mol) in ethanol (30 mL) was refluxed for 8 h. By cooling the solid product formed, filtered off and recrystallized from ethanol solvent gave compound **12e**.

White crystals (87%), m.p. 237–238 °C, ^1^H-NMR (400 MHz, DMSO), (δ, ppm), (*J*, Hz): 9.01 (brs, 1H, NHCH_2_CO); 8.26–8.28 (m, 1H, ArH); 7.89–7.91 (m, 1H, ArH); 7.78–7.86 (m, 2H, ArH); 7.30–7.35 (m, 3H, ArH); 7.16–7.20 (m, 2H, ArH); 7.08–7.12 (s, 1H, CH_3_-C=CH-C-CH_3_); 5.46–5.48 (d, *J* = 5.2, 2H, NHCH_2_CO); 4.89 (s, 2H, NCH_2_CO); 4.25 (s, 2H, CH_2_ph); 2.46 (s, 3H, CH_3_); 2.43 (s, 3H, CH_3_). ^13^C-NMR: 171.28 (C=O); 168.37 (C=O); 159.72 (C=O); 148.84 (C-CH_3_); 146.35 (C-Ar); 144.33 (C-CH_3_); 137.22 (C-Ar); 133.29 (CH-Ar); 131.67 (CH-Ar); 130.45 (C-Ar); 128.75 (2 CH-Ar); 128.40 (2 CH-Ar); 128.07 (C-Ar); 127.46 (CH-Ar);126.74 (CH-Ar); 125.31 (CH-Ar); 116.25 (CH_3_-C=CH-C-CH_3_); 55.25 (NCH_2_CO); 42.65 (NHCH_2_CO); 38.41 (CH_2_ph); 16.21 (CH_3_); 13.74 (CH_3_).

MS (MALDI, positive mode, matrix DHB) m/z: 452.50 (M + Na)^+^. Elemental analysis: calculated for C_24_H_23_N_5_O_3_ (429.48): % C, 67.12; % H, 5.40; % N, 16.31. Found: % C, 67.07; % H, 5.47; % N, 16.34.

## Biological assays

### Cytotoxicity of the synthesized compounds using MTT assay

MCF-7, HepG2 cancer cells and WISH normal cells were obtained from the National Cancer Institute in Cairo, Egypt, they were cultured in complete media of RPMI and DMEM, respectively at 5% carbon dioxide and 37 °C following standard tissue culture work. The cells were grown in “10% fetal bovine serum (FBS) and 1% penicillin–streptomycin” in 96-multiwell plate. All the synthesized compounds were screened for their cytotoxicity using 20 µL of MTT solution (Promega, USA) for 48 hours [46] using untreated and treated cells with concentrations of (0.01, 0.1, 1, 10, and 100 µM) for 48 h. The plate was cultured for 3 h. Percentage of cell viability was calculated following this equation:$100-(\mathbf{A}\mathrm{ Sample}/\mathbf{A}\mathrm{ Control})\mathrm{X}100$. An ELISA microplate reader was used to measure the absorbance at 690 nm to calculate the viability versus concentration, and the IC_50_ value using GraphPad prism software [47].

### EGFR inhibition

The most promising cytotoxic compounds were subjected to EGFR enzyme assay (BPS Bioscience Corporation catalog#40321) using ELISA kit (Enzyme-Linked Immunosorbent Assay) following manufacturer information [48]. The luminescence was measured with a microplate reader at 450 nm by ELISA Reader (PerkinElmer). Inhibition percentage was calculated following this equation: $100-[\frac{A control}{A treated}-Control)]$, IC_50_ was determined using GraphPad prism7 using inhibition curves at five different concentrations of each compound.

### Flow cytometry using annexin V/PI staining

MDA-MB-231 cells were incubated overnight in 6-well culture plates (3–5 × 10^5^ cells/well) and then treated with the IC_50_ values for 48 h with compound **12d**. After that, the cells were incubated in a 100 µL solution of Annexin binding buffer "25 mM CaCl_2_, 1.4 M NaCl, and 0.1 M Hepes/NaOH, pH 7.4" in the dark for 30 min with "Annexin V-FITC solution (1:100) and propidium iodide (PI) at a concentration equivalent to 10 g/mL." The labeled cells were then extracted using the Cytoflex FACS machine. CytExpert software was used to analyze the data [47, 49, 50].

### Molecular docking study

Molecular modeling studies were carried out using Chimera-UCSF and AutoDock Vina on Linux-based systems at the laboratory of Drug Design and Discovery, Suez Canal University. Proteins and compounds structures were prepared and optimized using Maestro, then binding sites inside proteins were determined using grid-box dimensions around the co-crystallized ligands. The investigated compounds were docked against the protein structures of EGFR (PDB = 1M17) using AutoDock Vina software following routine work [51, 52]. Vina was used to improve protein and ligand structures and to favor them energetically. Binding activities interpreted molecular docking results in terms of binding energy and ligand-receptor interactions. The visualization was then done with Chimera. ADME pharmacokinetics study was carried out using web-based software “Molsoft” as previously utilized in Youssef et al. [53].

## Conclusion

In this study, we synthesized twenty-nine new phthalazinone derivatives starting from 4-Benzyl-2H-phthalazin-1-one **(2)** and their chemical structure were elucidated via different analytical and spectroscopic methods. The cytotoxicity of the synthesized compounds was tested using MTT assay, as well as apoptosis-induction through EGFR inhibition. Compounds **11d**, **12c** and **12d** exhibited potent cytotoxic activities with IC_50_ values of 0.92, 1.89 and 0.57 μM against MDA-MB-231 cells compared to Erlotinib (IC_50_ = 1.02 μM). Interestingly compound **12d** exhibited promising potent EGFR inhbition with an IC_50_ value 21.4 nM compared to Erlotinib (IC_50_ = 80 nM). For apoptosis, compounds **12d** induced apoptosis in MDA-MB-231 cells by 64.4-fold (42.5% compared to 0.66 for the control), Hence, this compound may serve as a potential target-oriented anti-breast cancer agent (Additional file 1).

## Supplementary Information

**Additional file 1.** Characterization analyses for the synthesized compounds are provided as a Additional file. **Figure S1.** The 1H-NMR spectrum of compound 6a. **Figure S2.** The 13C-NMR spectrum of compound 6a.**Figure S3.** The 1H-NMR spectrum of compound 6b. **Figure S4.** The 13C-NMR spectrum of compound 6b. **Figure S5.** The 1H-NMR spectrum of compound 6c. **Figure S6.** The 13C-NMR spectrum of compound 6c. **Figure S7.** The 1H-NMR spectrum of compound 6d. **Figure S8.** The 13C-NMR spectrum of compound 6d. **Figure S9.** The 1H-NMR spectrum of compound 6e. **Figure S10.** The 13C-NMR spectrum of compound 6e. **Figure S11.** The 1H-NMR spectrum of compound 6f. **Figure S12.** The 13C-NMR spectrum of compound 6f. **Figure S13.** The 1H-NMR spectrum of compound 6g. **Figure S14.** The 13C-NMR spectrum of compound 6g. **Figure S15.** The 1H-NMR spectrum of compound 6h. **Figure S16.** The 13C-NMR spectrum of compound 6h. **Figure S17.** The 1H-NMR spectrum of compound 7a. **Figure S18.** The 13C-NMR spectrum of compound 7a. **Figure S19.** The 1H-NMR spectrum of compound 7c. **Figure S20.** The 13C-NMR spectrum of compound 7c. **Figure S21.**. The 1H-NMR spectrum of compound 7d. **Figure S22.** The 13C-NMR spectrum of compound 7d. **Figure S23.** The 1H-NMR spectrum of compound 8a. **Figure S24.** The 13C-NMR spectrum of compound 8a. **Figure S25.** The 1H-NMR spectrum of compound 10a. **Figure S26.** The 13C-NMR spectrum of compound 10a. **Figure S27.** The 1H-NMR spectrum of compound 10b. **Figure S28.** The 13C-NMR spectrum of compound 10b. **Figure S29.** The 1H-NMR spectrum of compound 10c. **Figure S30.** The 13C-NMR spectrum of compound 10c . **Figure S31.** The 1H-NMR spectrum of compound 10d. **Figure S32.** The 13C-NMR spectrum of compound 10d. **Figure S33.** The 1H-NMR spectrum of compound 10e. **Figure S34.**. The 13C-NMR spectrum of compound 10e. **Figure S35.** The 1H-NMR spectrum of compound 10f . **Figure S36.** The 13C-NMR spectrum of compound 10f. **Figure S37.** The 1H-NMR spectrum of compound 10h. **Figure S38.** The 13C-NMR spectrum of compound 10h. **Figure S39.** The 1H-NMR spectrum of compound 11a. **Figure S40.** The 13C-NMR spectrum of compound 11a. **Figure S41.** The 1H-NMR spectrum of compound 11b. **Figure S42.**. The 13C-NMR spectrum of compound 11b. **Figure S43.** The 1H-NMR spectrum of compound 12a.**Figure S44.** The 1H-NMR spectrum of compound 12b. **Figure S45.** The 1H-NMR spectrum of compound 12c. **Figure S46.** The 1H-NMR spectrum of compound 12d. **Figure S47.** The 13C-NMR spectrum of compound 12d. **Figure S48.** The 1H-NMR spectrum of compound 12e.

## Data Availability

All data and analyses are available from the corresponding author on reasonable request.

## Abbreviations

EGFR: Epidermal growth factor receptor
RT-PCR: Reverse transcription polymerase
MTT: 3-(4,5-Dimethylthiazolyl-2)-2,5-diphenyltetrazolium bromide
SD: Standard deviation
IC_50_: Half-maximal inhibitory concentration
MCF-7 and MDA-MB-231: Breast cancer cell lines
WISH: Normal cells
G2/M, S, G1, G0: Cell cycle phases
