# Supplementary material for: Synthesis of phthalazine-based derivatives as selective anti-breast cancer agents through EGFR-mediated apoptosis: in vitro and in silico studies
Source: BMC Chem. 2023 Jul 27;17(1):90. doi: 10.1186/s13065-023-00995-2 (PMC10375784; doi:10.1186/s13065-023-00995-2)
Supplement: Supplementary file 1 — Additional file 1. Characterization analyses for the synthesized compounds are provided as a Additional file. Figure S1. The 1H-NMR spectrum of compound 6a. Figure S2. The 13C-NMR spectrum of compound 6a.Figure S3. The 1H-NMR spectrum of compound 6b. Figure S4. The 13C-NMR spectrum of compound 6b. Figure S5. The 1H-NMR spectrum of compound 6c. Figure S6. The 13C-NMR spectrum of compound 6c. Figure S7. The 1H-NMR spectrum of compound 6d. Figure S8. The 13C-NMR spectrum of compound 6d. Figure S9. The 1H-NMR spectrum of compound 6e. Figure S10. The 13C-NMR spectrum of compound 6e. Figure S11. The 1H-NMR spectrum of compound 6f. Figure S12. The 13C-NMR spectrum of compound 6f. Figure S13. The 1H-NMR spectrum of compound 6g. Figure S14. The 13C-NMR spectrum of compound 6g. Figure S15. The 1H-NMR spectrum of compound 6h. Figure S16. The 13C-NMR spectrum of compound 6h. Figure S17. The 1H-NMR spectrum of compound 7a. Figure S18. The 13C-NMR spectrum of compound 7a. Figure S19. The 1H-NMR spectrum of compound 7c. Figure S20. The 13C-NMR spectrum of compound 7c. Figure S21.. The 1H-NMR spectrum of compound 7d. Figure S22. The 13C-NMR spectrum of compound 7d. Figure S23. The 1H-NMR spectrum of compound 8a. Figure S24. The 13C-NMR spectrum of compound 8a. Figure S25. The 1H-NMR spectrum of compound 10a. Figure S26. The 13C-NMR spectrum of compound 10a. Figure S27. The 1H-NMR spectrum of compound 10b. Figure S28. The 13C-NMR spectrum of compound 10b. Figure S29. The 1H-NMR spectrum of compound 10c. Figure S30. The 13C-NMR spectrum of compound 10c . Figure S31. The 1H-NMR spectrum of compound 10d. Figure S32. The 13C-NMR spectrum of compound 10d. Figure S33. The 1H-NMR spectrum of compound 10e. Figure S34.. The 13C-NMR spectrum of compound 10e. Figure S35. The 1H-NMR spectrum of compound 10f . Figure S36. The 13C-NMR spectrum of compound 10f. Figure S37. The 1H-NMR spectrum of compound 10h. Figure S38. The 13C-NMR spectrum of compound 10h. Figure S39. The 1H-NMR spectrum of comp [file 13065_2023_995_MOESM1_ESM.docx]

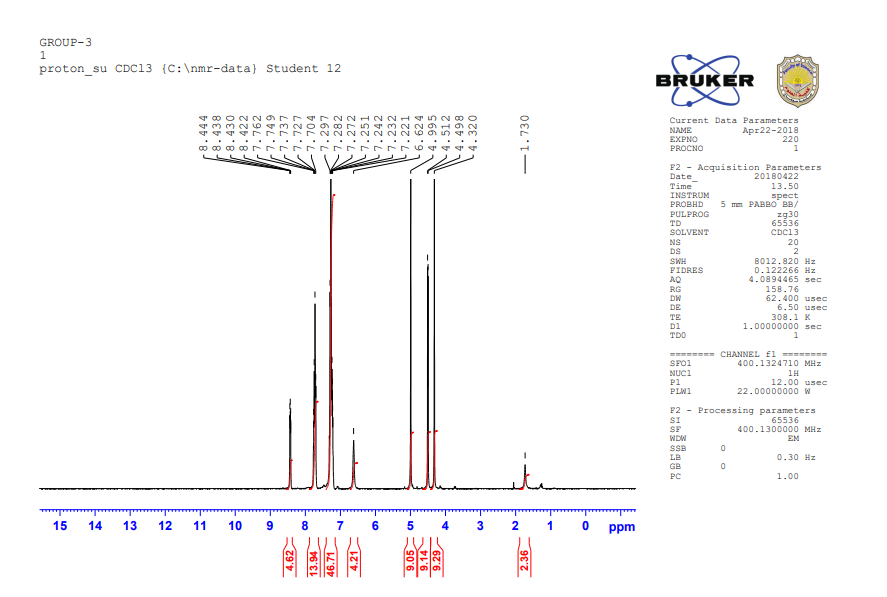
**Figure S1**. The ^1^H-NMR spectrum of compound **6a**

**Figure S2**. The ^13^C-NMR spectrum of compound **6a**


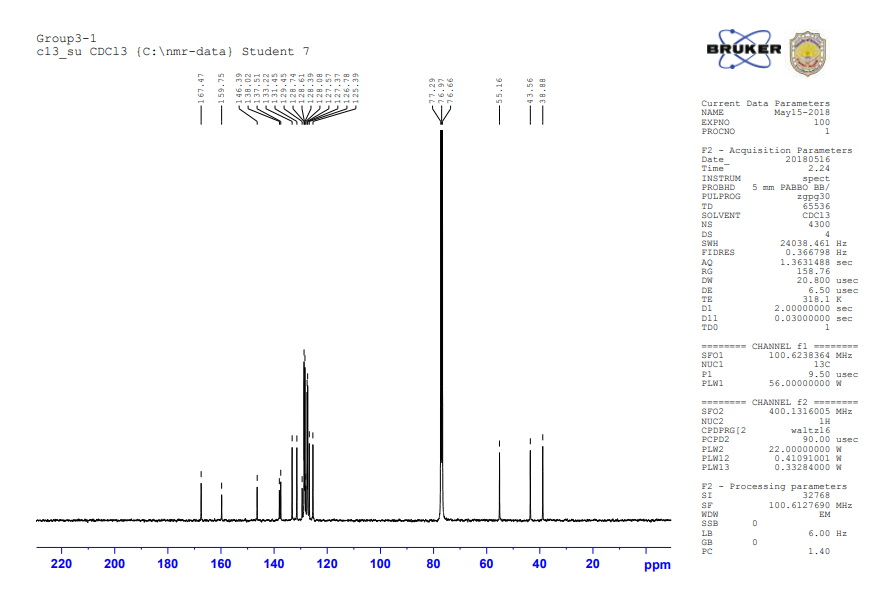

**
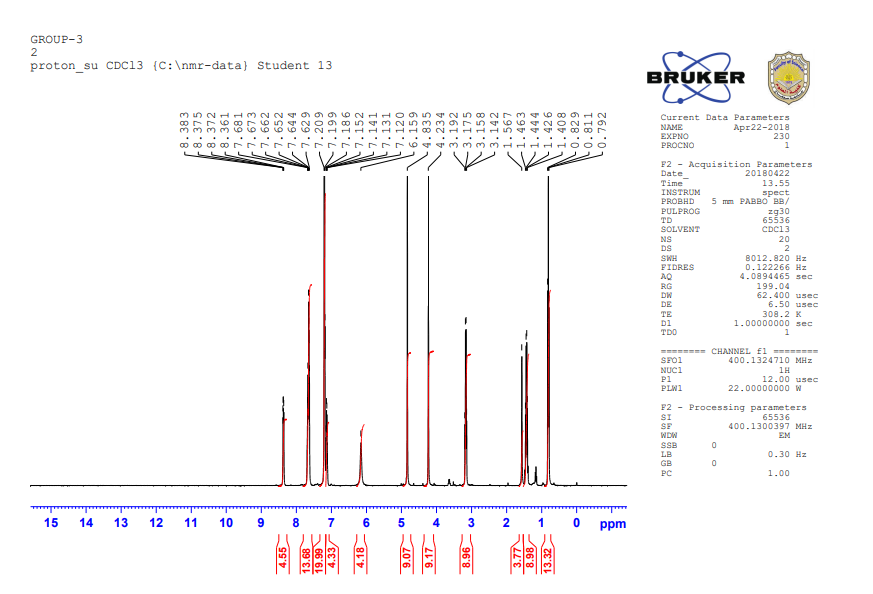
Figure S3**. The ^1^H-NMR spectrum of compound **6b**

**
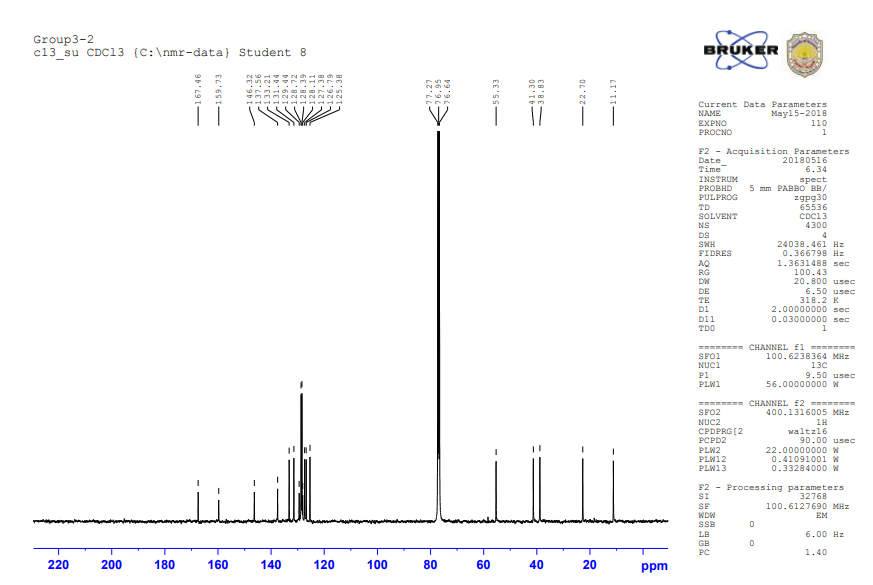
Figure S4**. The ^13^C-NMR spectrum of compound **6b**

**
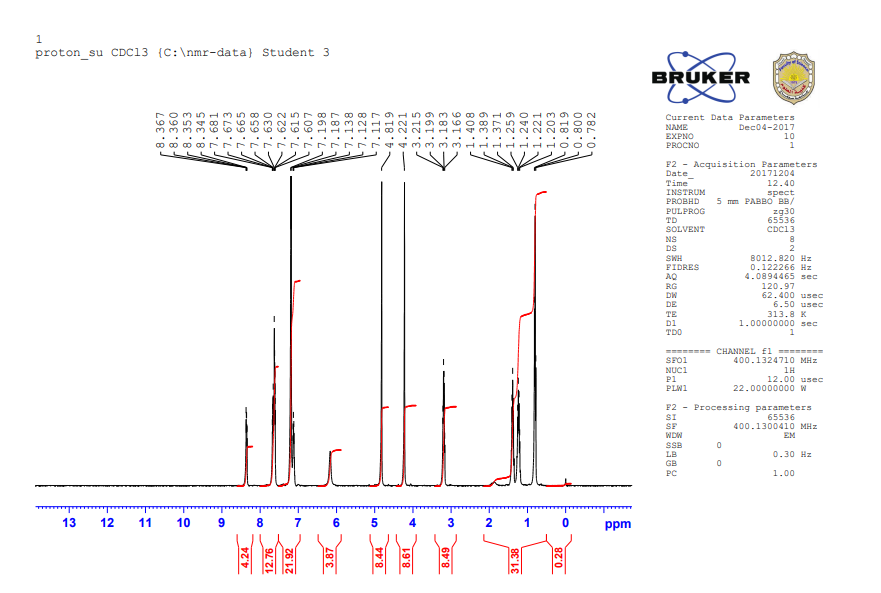
Figure S5**. The ^1^H-NMR spectrum of compound **6c**

**Figure S6**. The ^13^C-NMR spectrum of compound **6c**

**
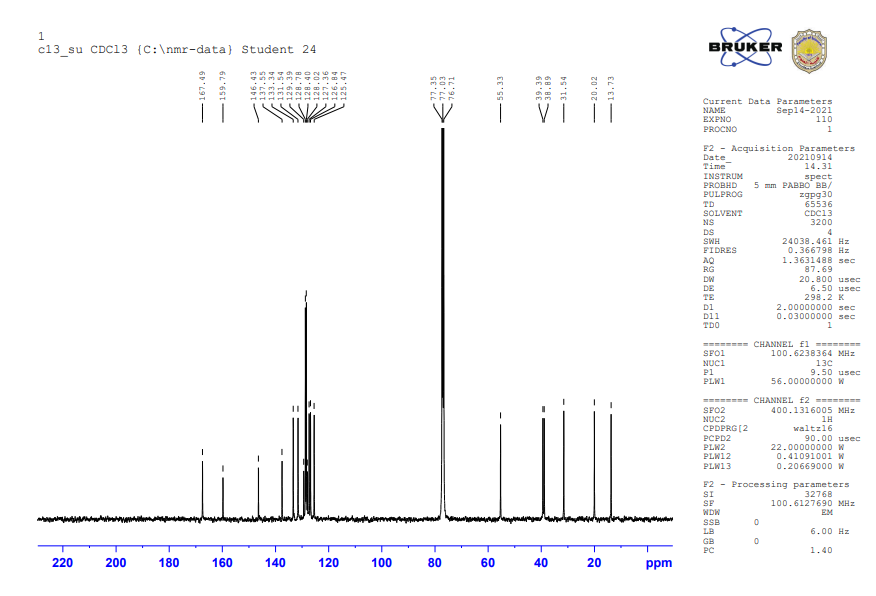
**


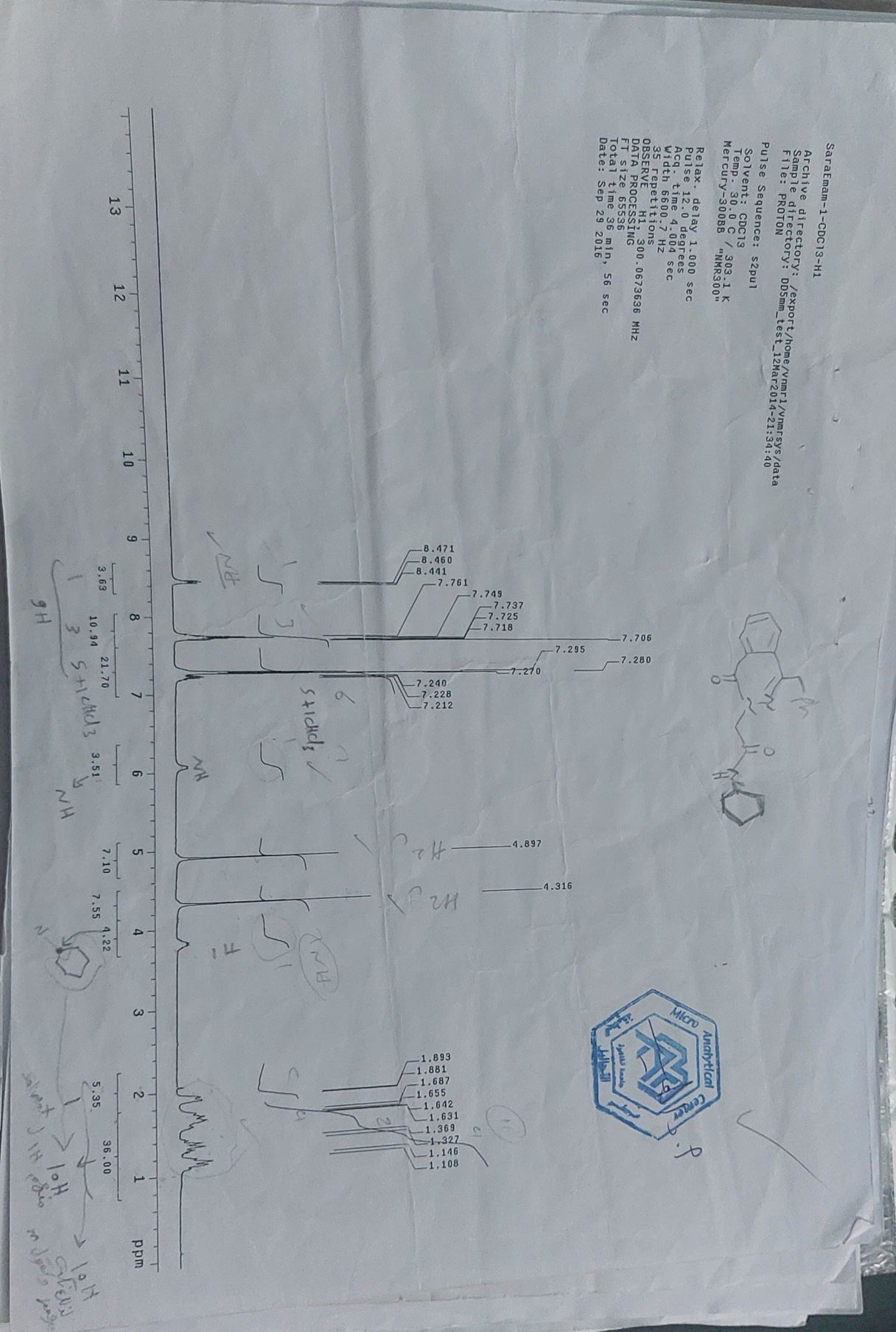


**Figure S7**. The ^1^H-NMR spectrum of compound **6d**

**
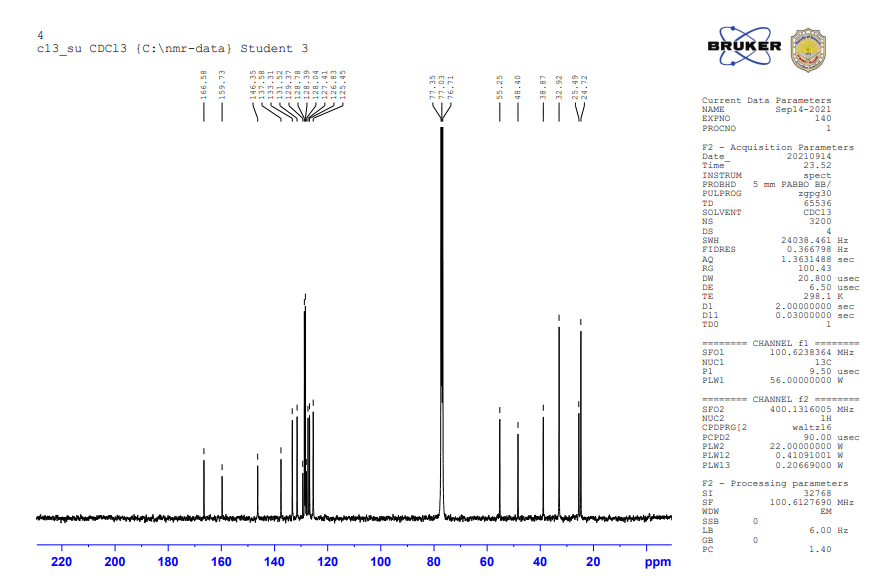
Figure S8**. The ^13^C-NMR spectrum of compound **6d**


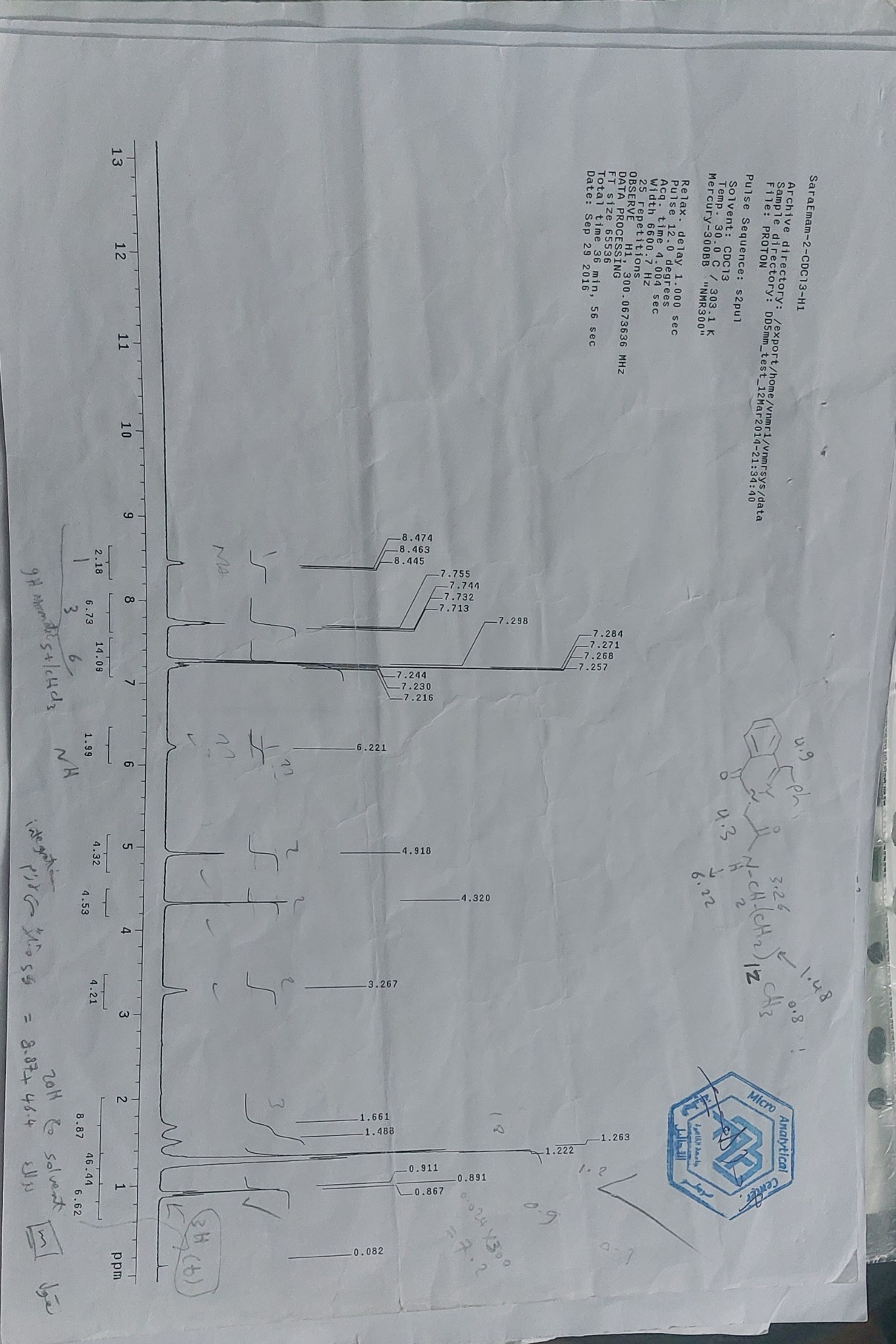


**Figure S9**. The ^1^H-NMR spectrum of compound **6e**

**
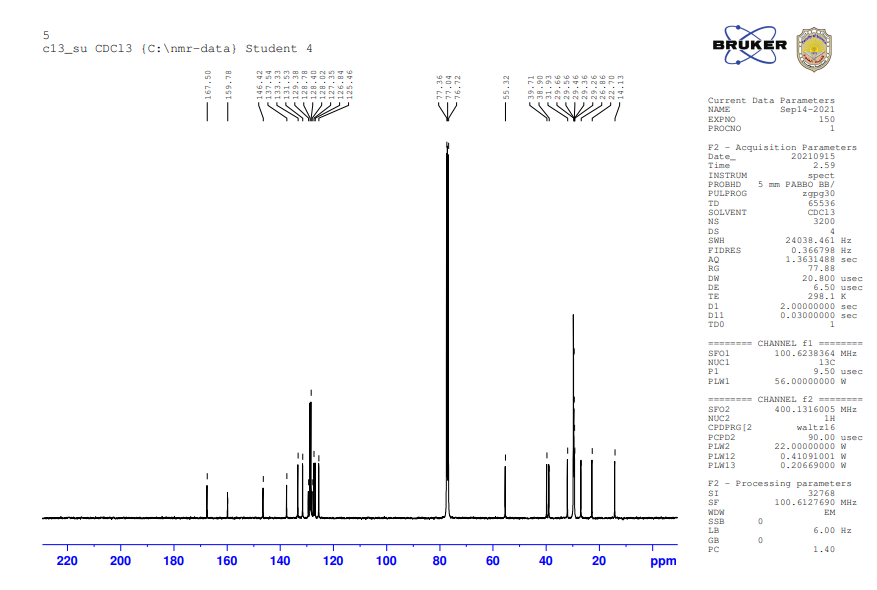
Figure S10**. The ^13^C-NMR spectrum of compound **6e**

**Figure S11**. The ^1^H-NMR spectrum of compound **6f**

**
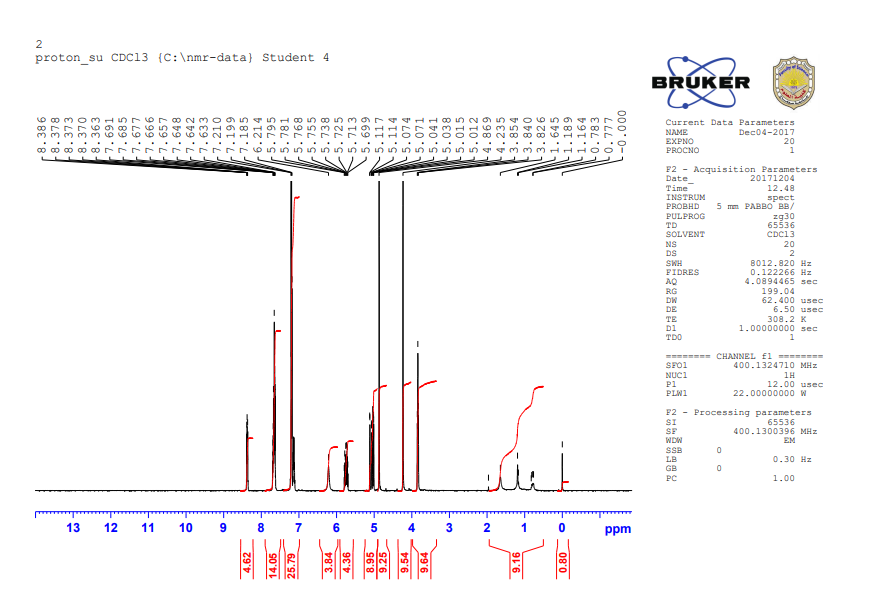
**

**
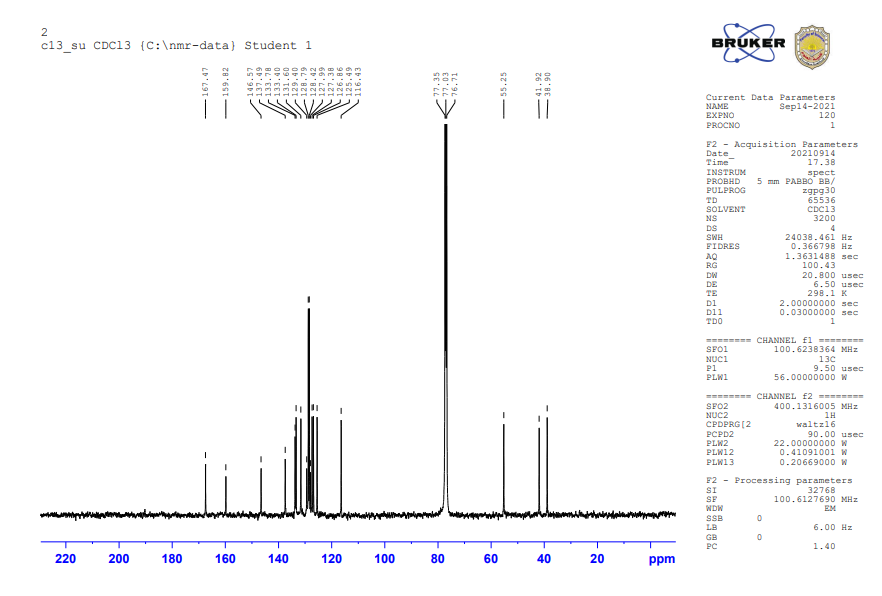
Figure S12**. The ^13^C-NMR spectrum of compound **6f**

**
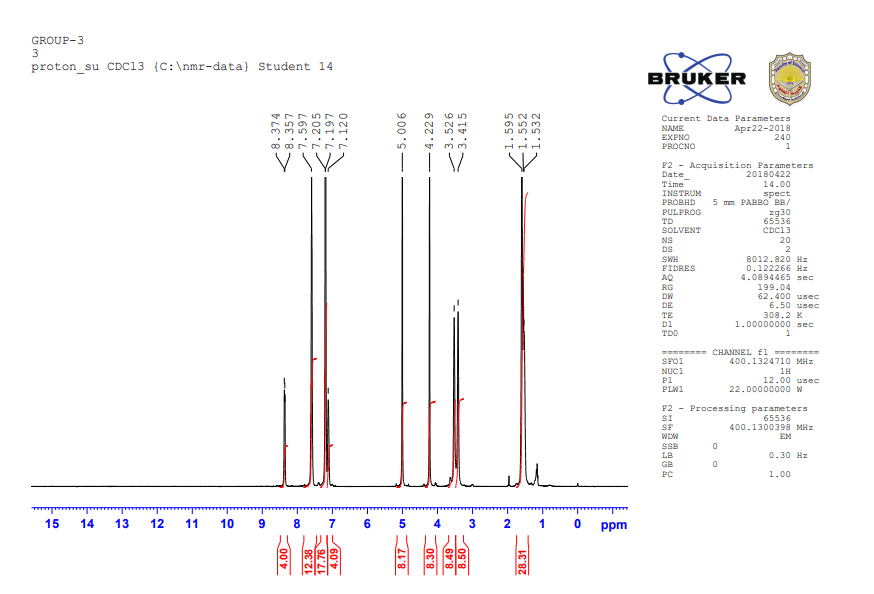
Figure S13**. The ^1^H-NMR spectrum of compound **6g**

**
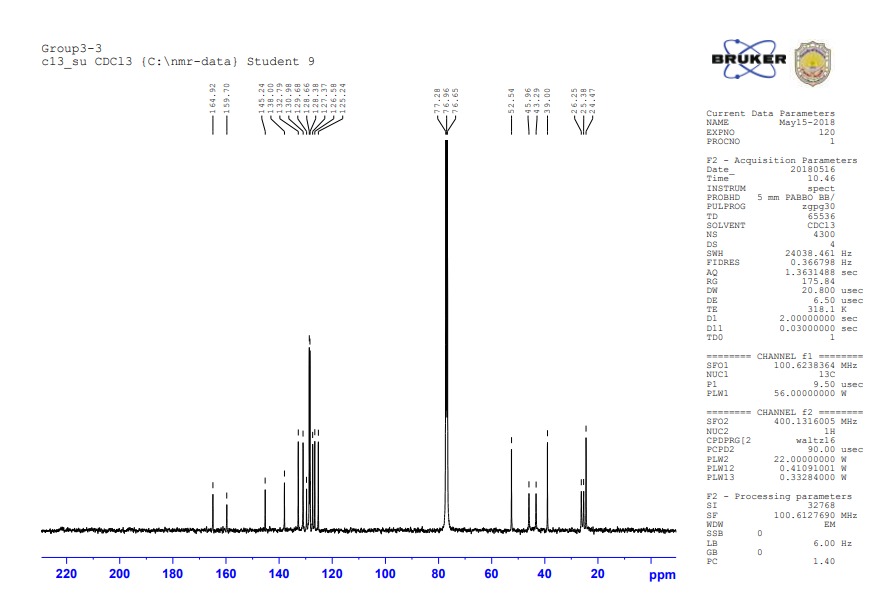
Figure S14**. The ^13^C-NMR spectrum of compound **6g**

**Figure S15**. The ^1^H-NMR spectrum of compound **6h**

**
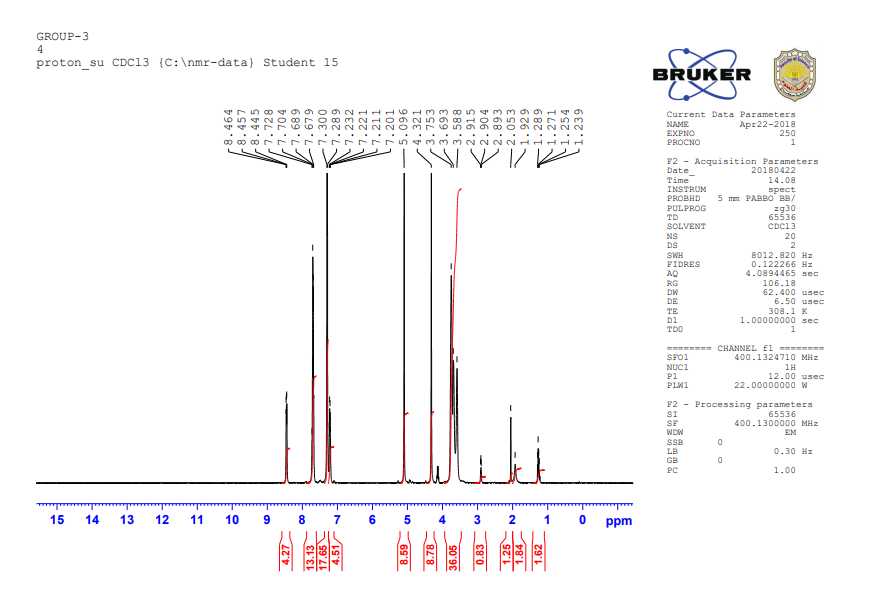
**

**
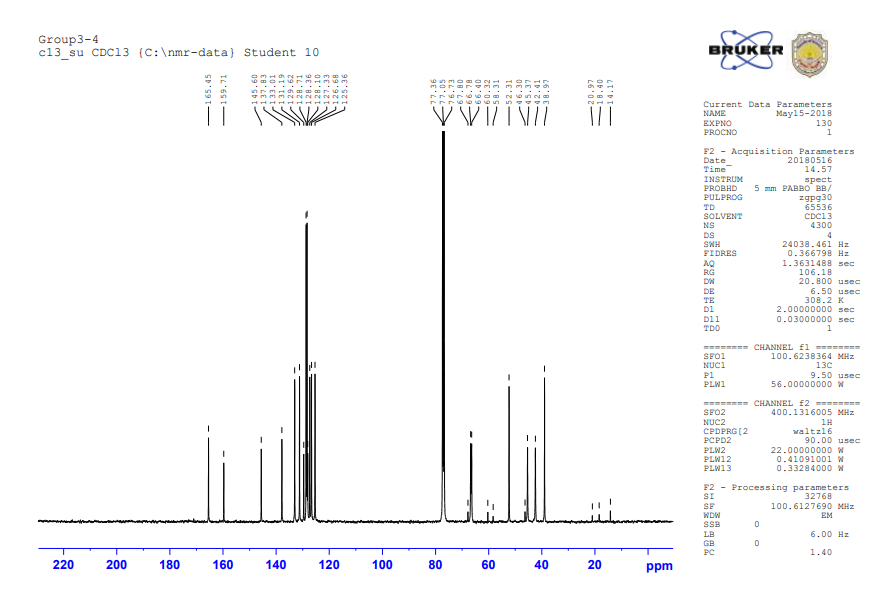
Figure S16**. The ^13^C-NMR spectrum of compound **6h**

**
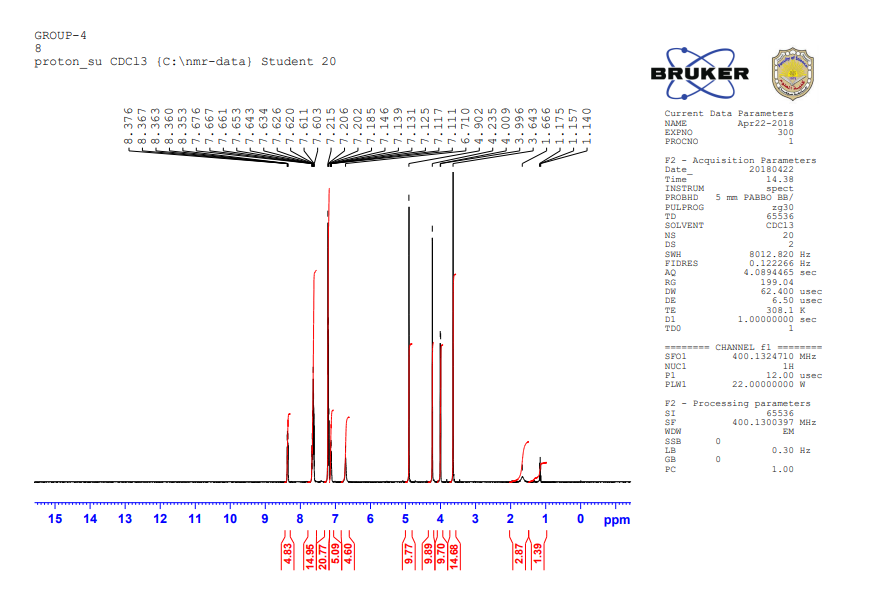
Figure S17**. The ^1^H-NMR spectrum of compound **7a**

**
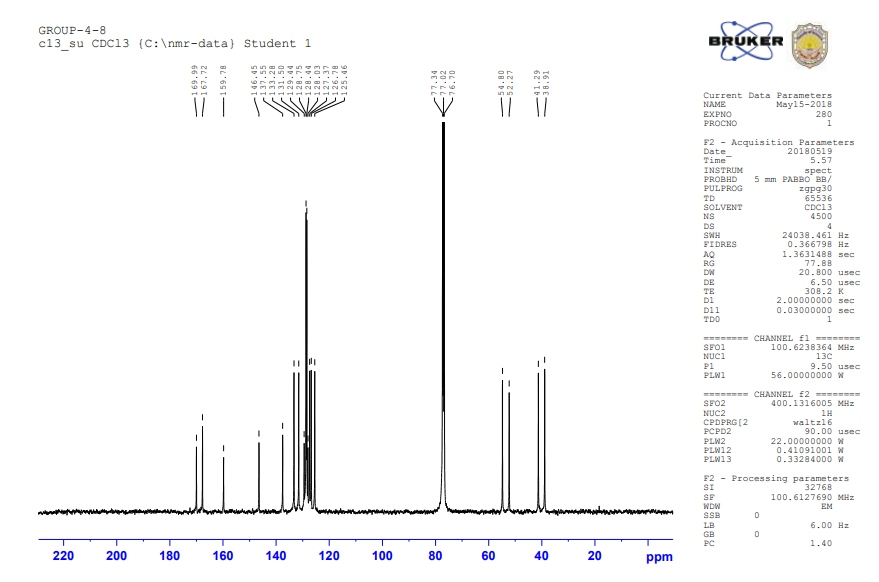
 Figure S18**. The ^13^C-NMR spectrum of compound **7a**

**Figure S19**. The ^1^H-NMR spectrum of compound **7c**


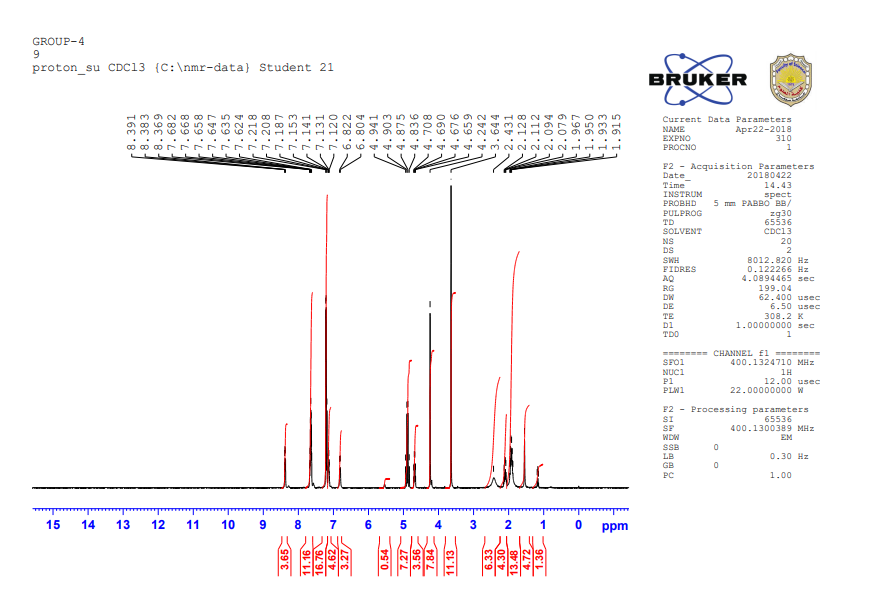

**Figure S20**. The ^13^C-NMR spectrum of compound **7c**

**
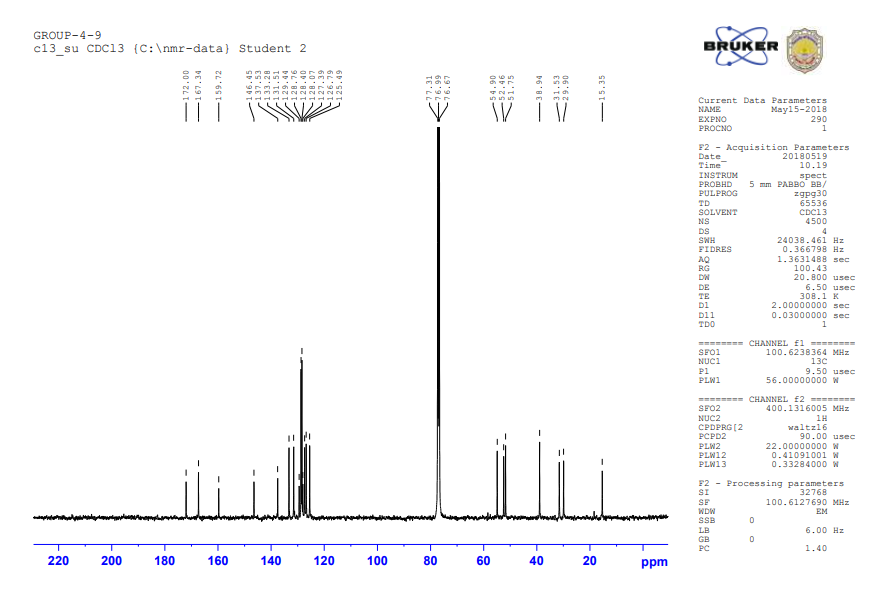
**


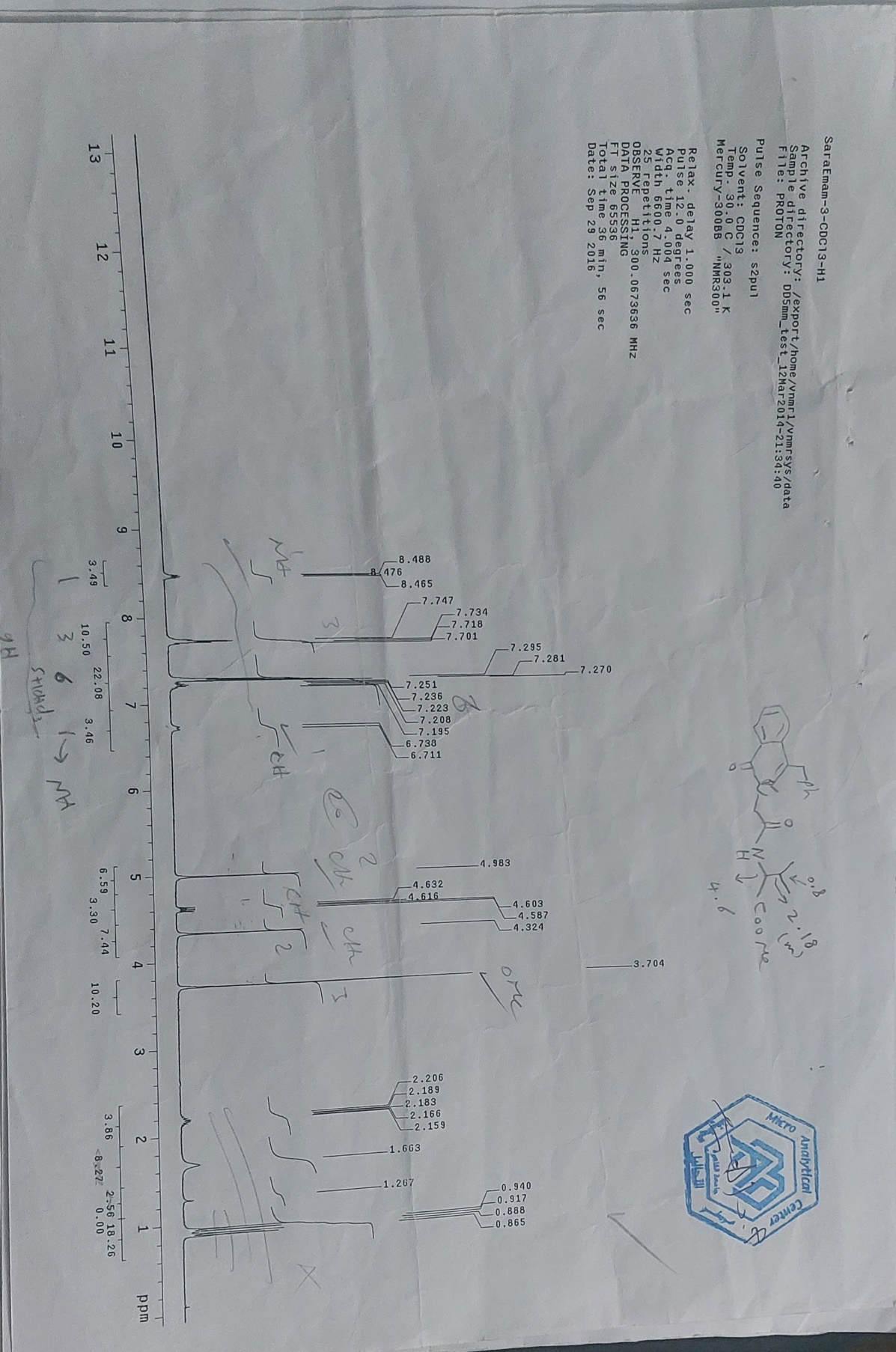


**Figure S21**. The ^1^H-NMR spectrum of compound **7d**

**
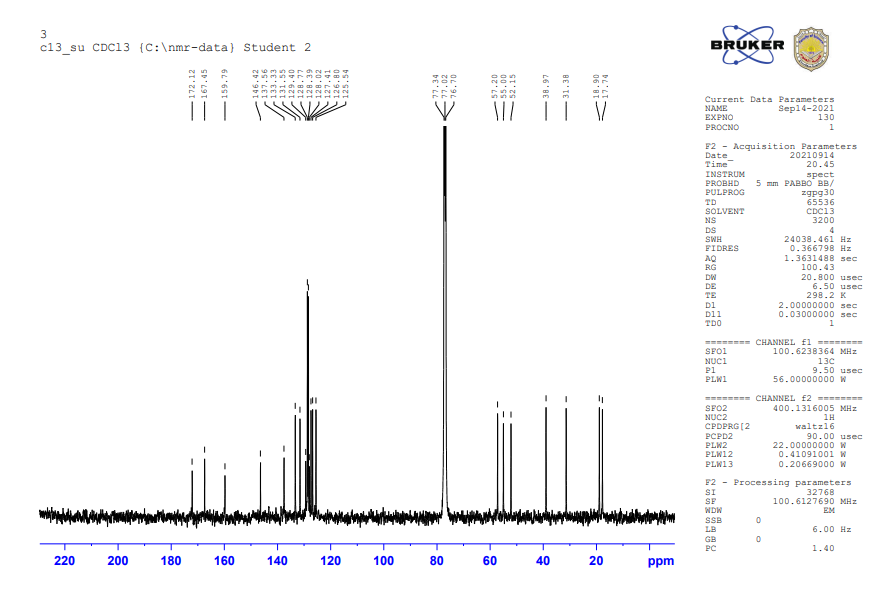
Figure S22**. The ^13^C-NMR spectrum of compound **7d**

**Figure S23**. The ^1^H-NMR spectrum of compound **8a**

**
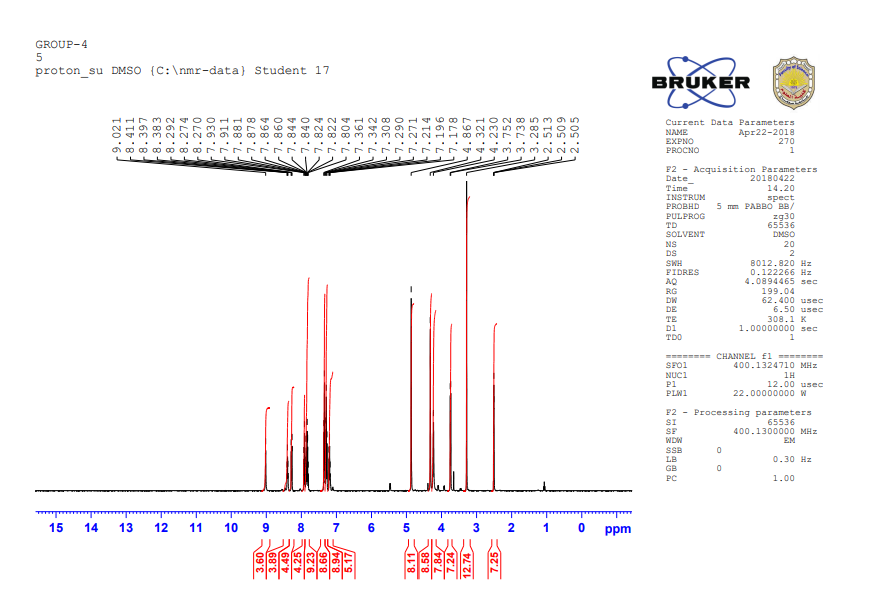
**

**
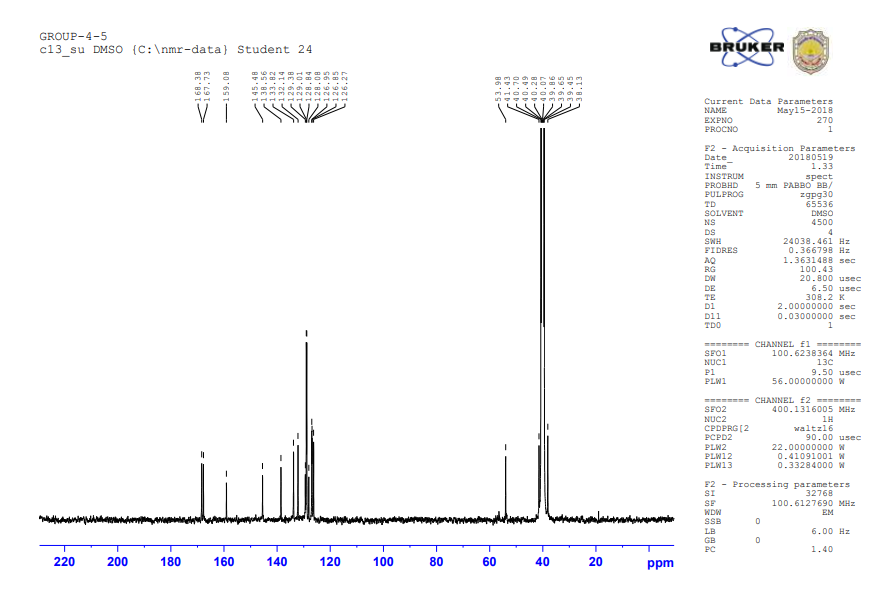
Figure S24**. The ^13^C-NMR spectrum of compound **8a**

**Figure S25**. The ^1^H-NMR spectrum of compound **10a**

**
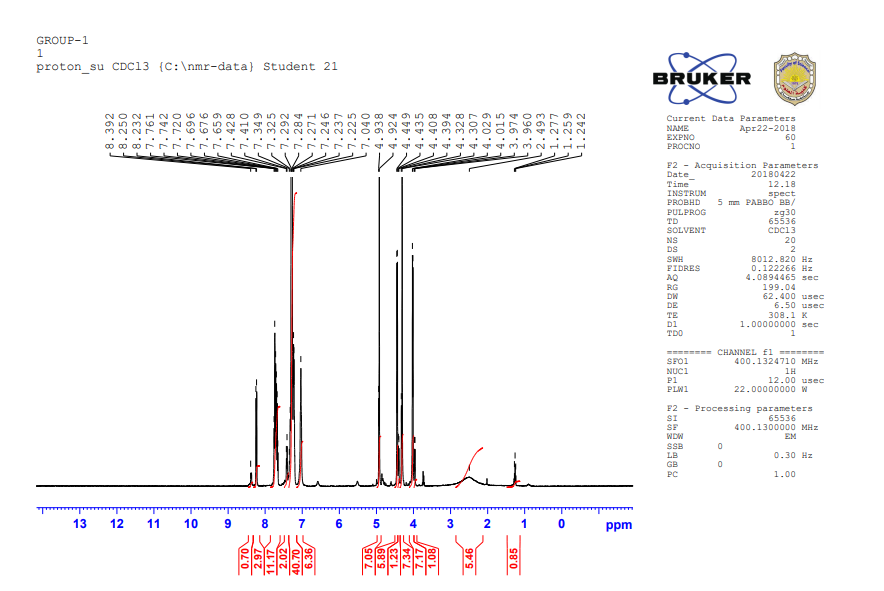
**

**Figure S26**. The ^13^C-NMR spectrum of compound **10a**

**
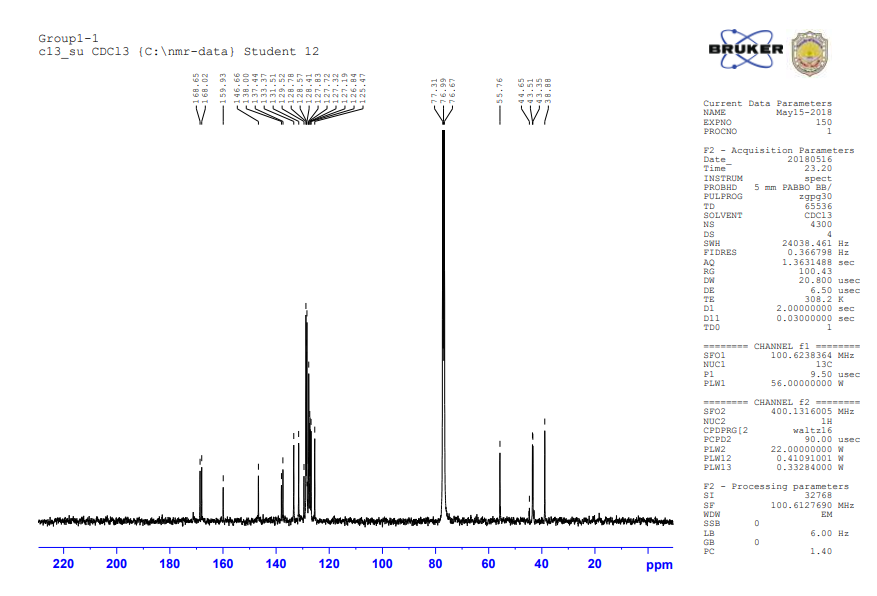
**

**Figure S27**. The ^1^H-NMR spectrum of compound **10b**

**
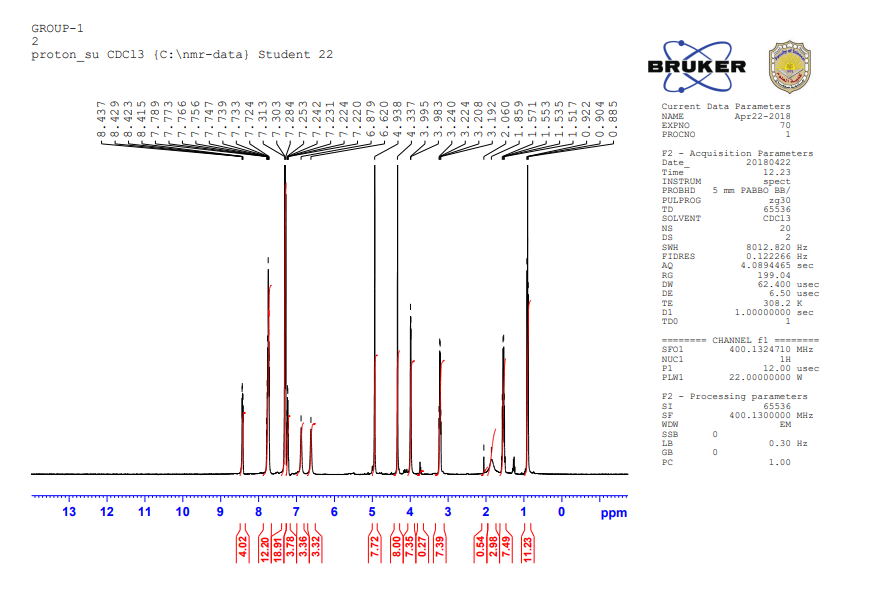
**

**Figure S28**. The ^13^C-NMR spectrum of compound **10b**

**
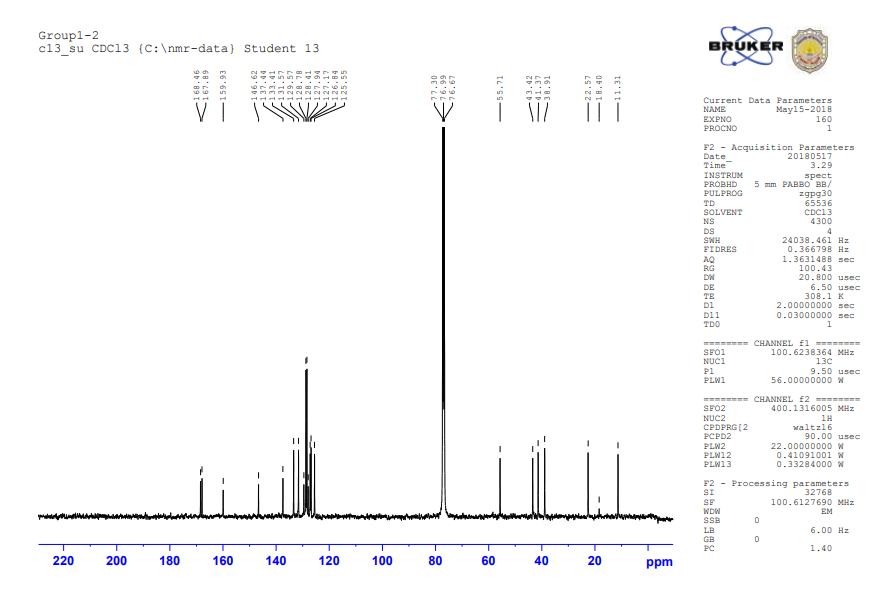
**

**
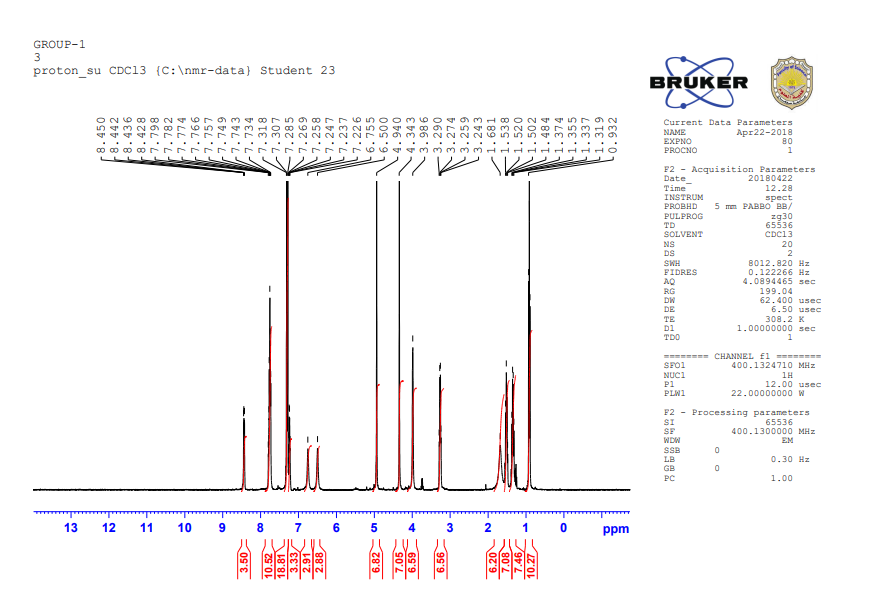
Figure S29**. The ^1^H-NMR spectrum of compound **10c**

**Figure S30**. The ^13^C-NMR spectrum of compound **10c**

**
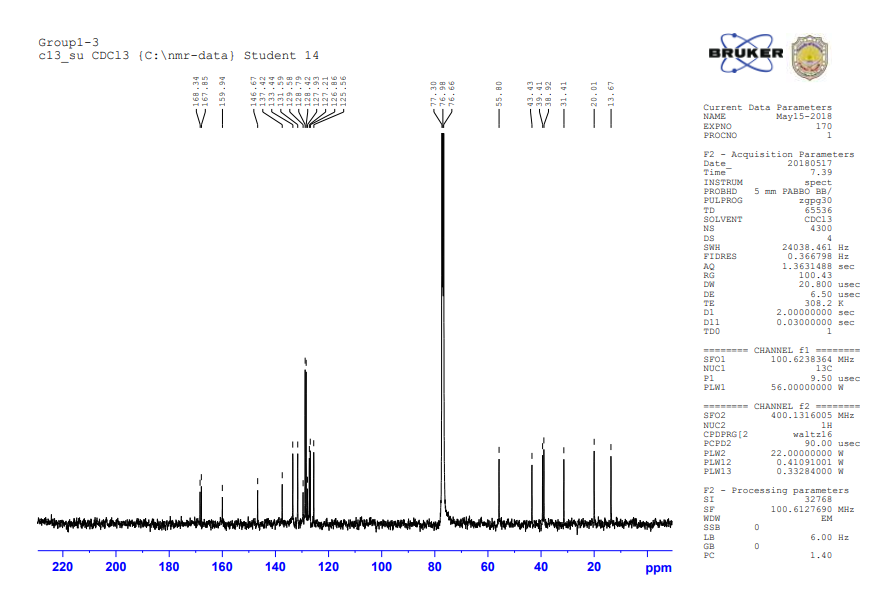
**

**
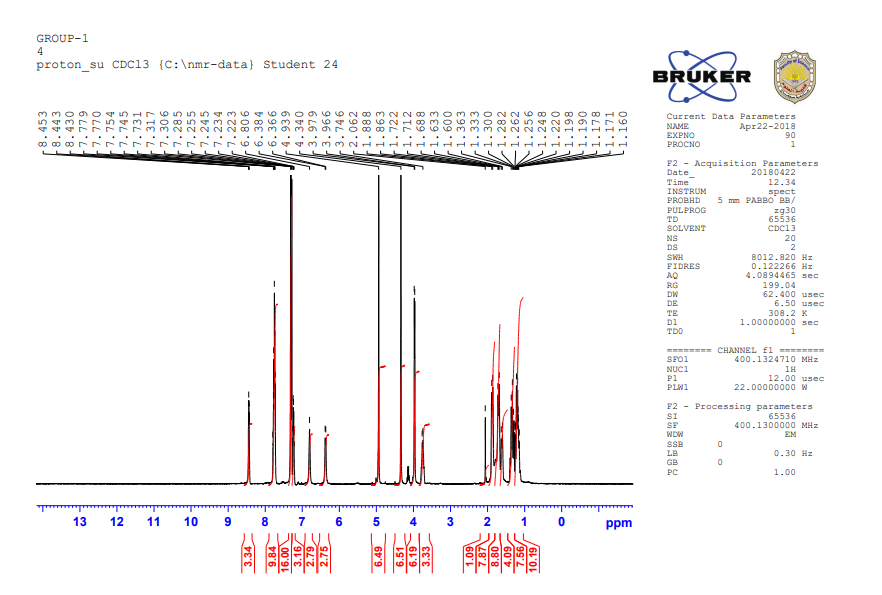
Figure S31**. The ^1^H-NMR spectrum of compound **10d**

**
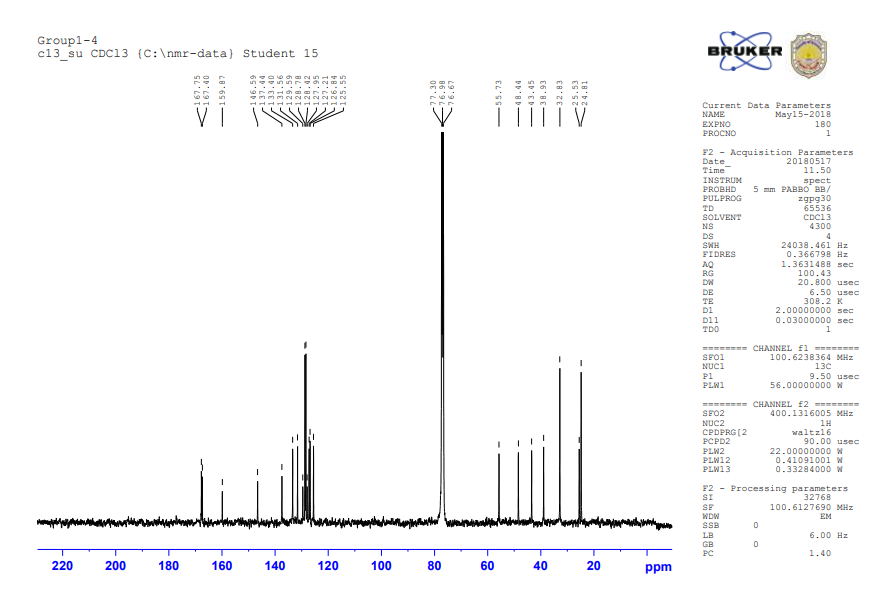
Figure S32**. The ^13^C-NMR spectrum of compound **10d**

**
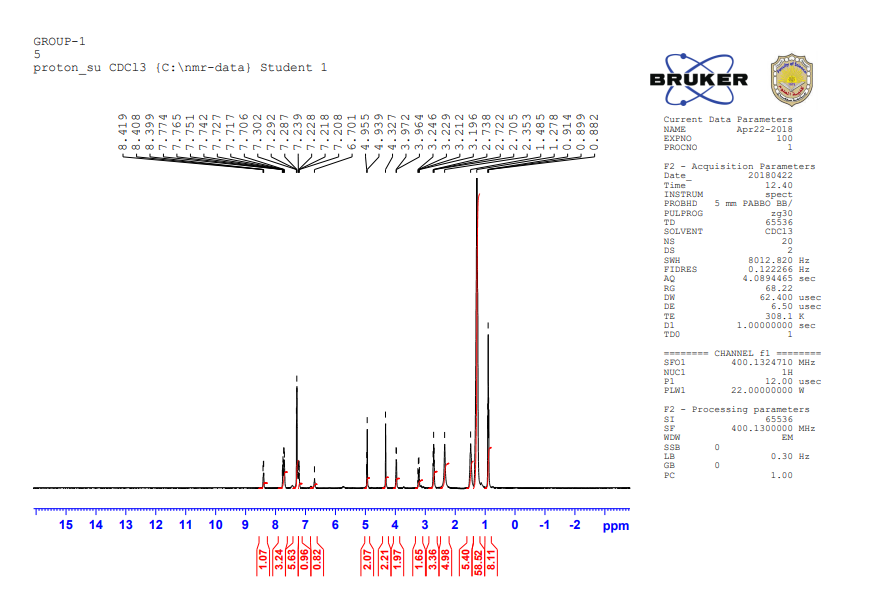
Figure S33**. The ^1^H-NMR spectrum of compound **10e**

**
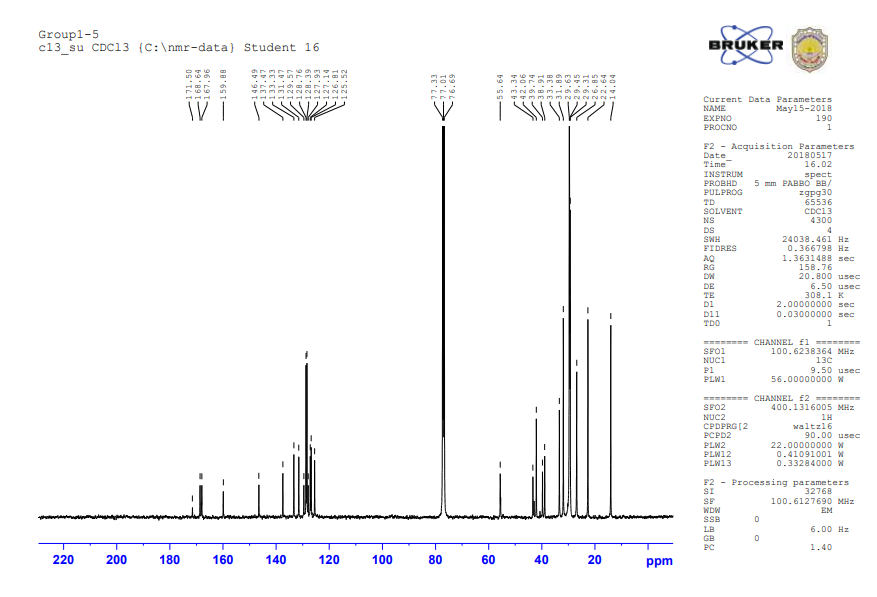
Figure S34**. The ^13^C-NMR spectrum of compound **10e**

**Figure S35**. The ^1^H-NMR spectrum of compound **10f**

**
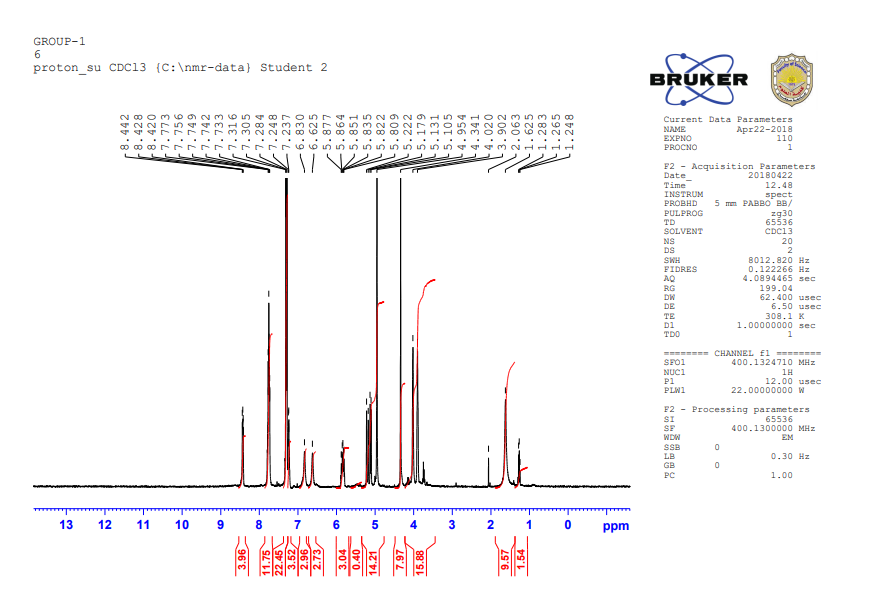
**

**Figure S36**. The ^13^C-NMR spectrum of compound **10f**

**
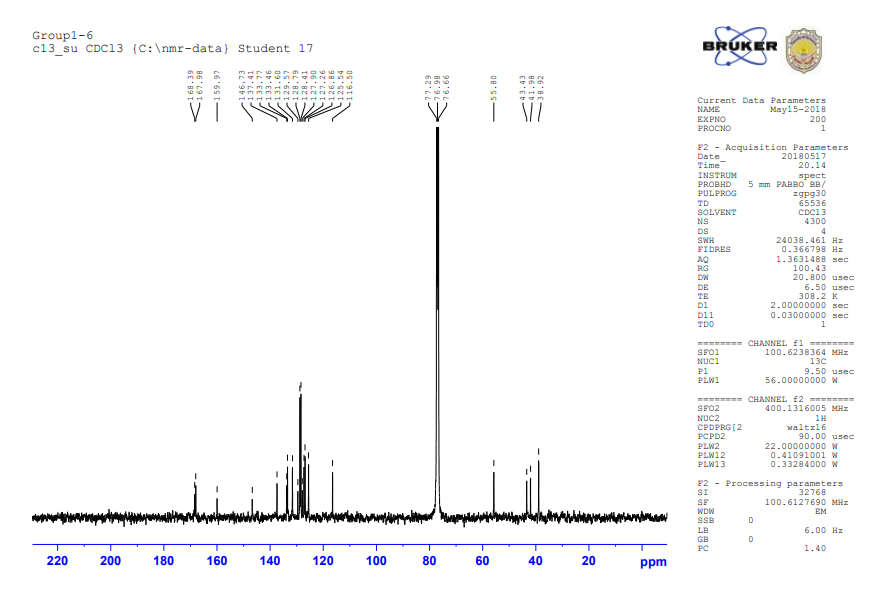
**

**
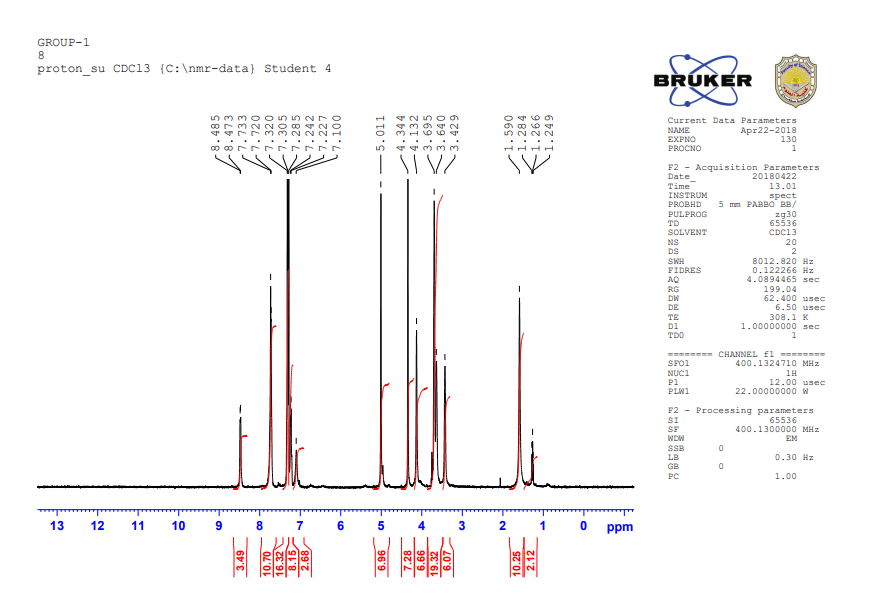
Figure S37**. The ^1^H-NMR spectrum of compound **10h**

**
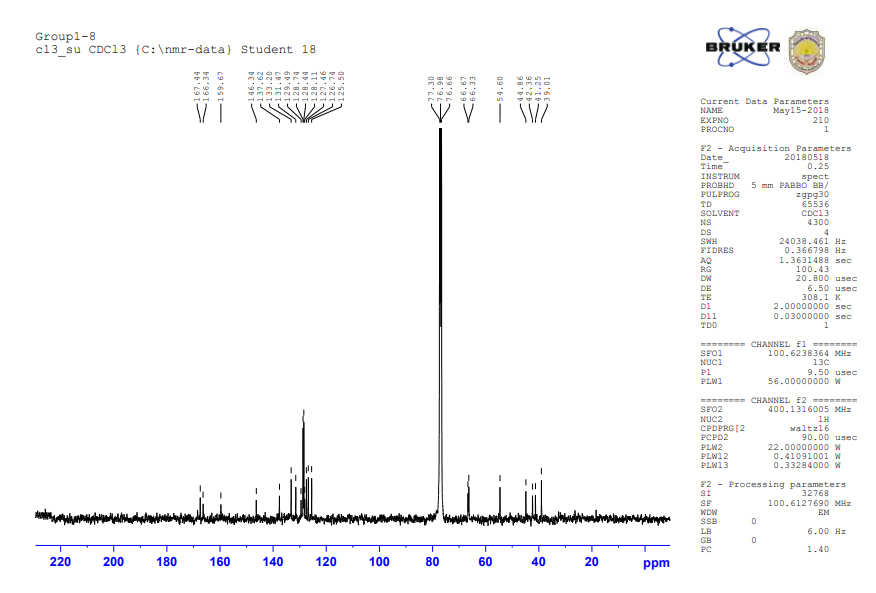
Figure S38**. The ^13^C-NMR spectrum of compound **10h**

**
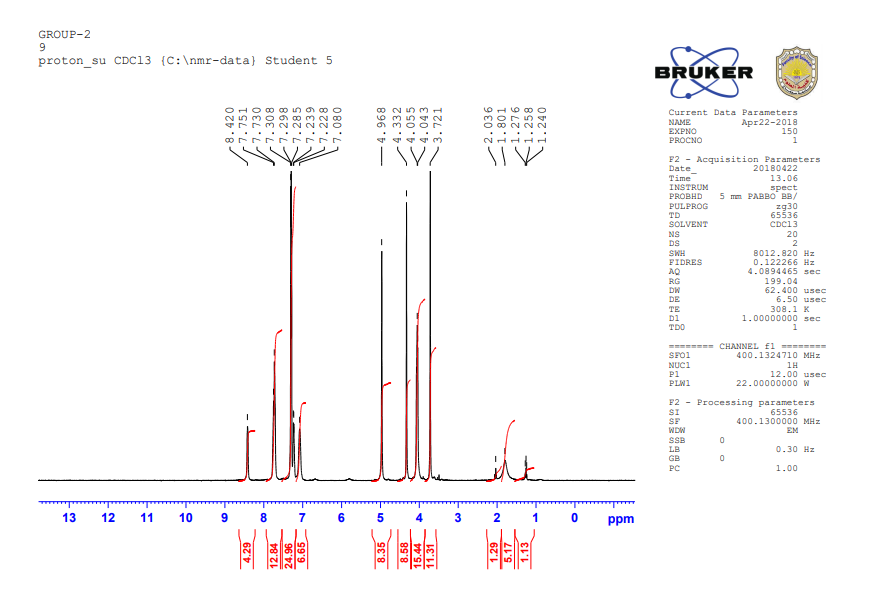
Figure S39**. The ^1^H-NMR spectrum of compound **11a**

**Figure S40**. The ^13^C-NMR spectrum of compound **11a**

**
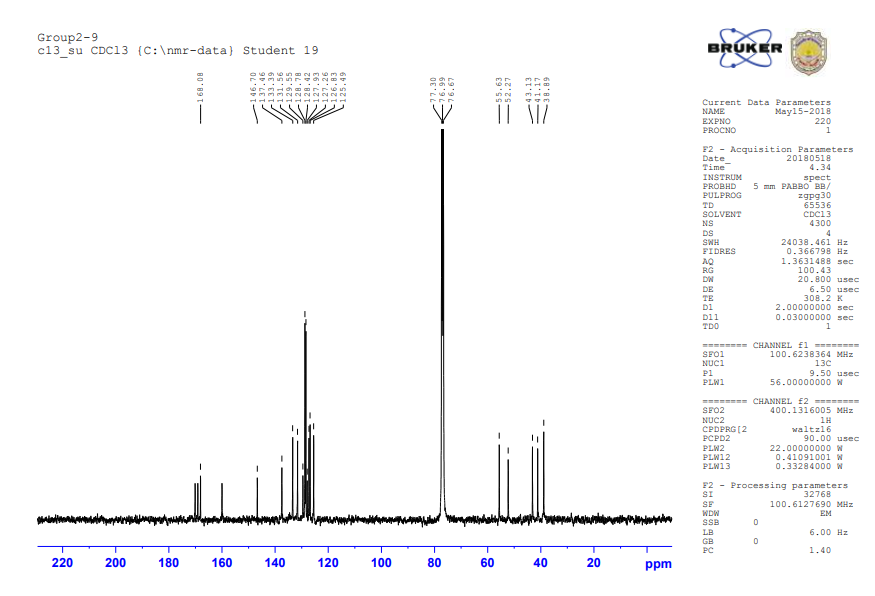
**

**Figure S41**. The ^1^H-NMR spectrum of compound **11b**

**
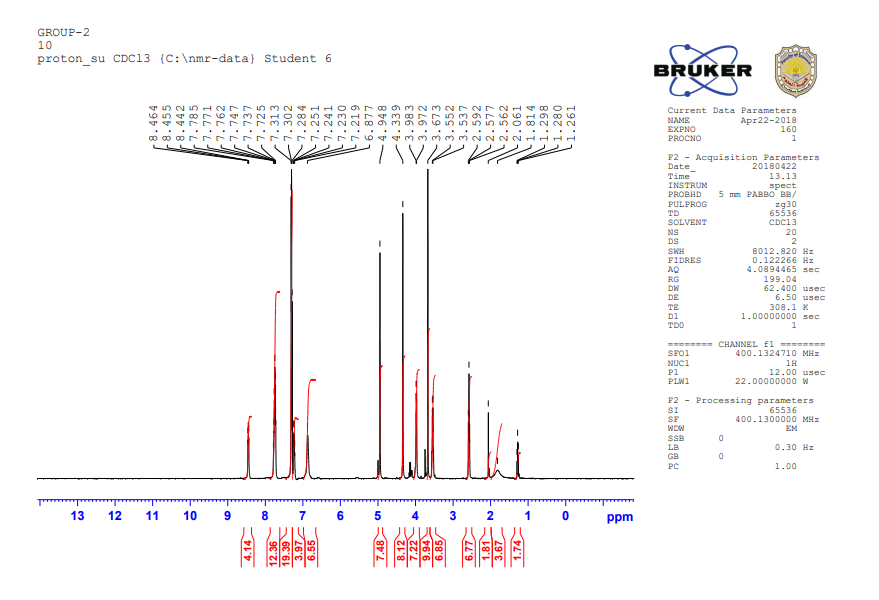
**

**
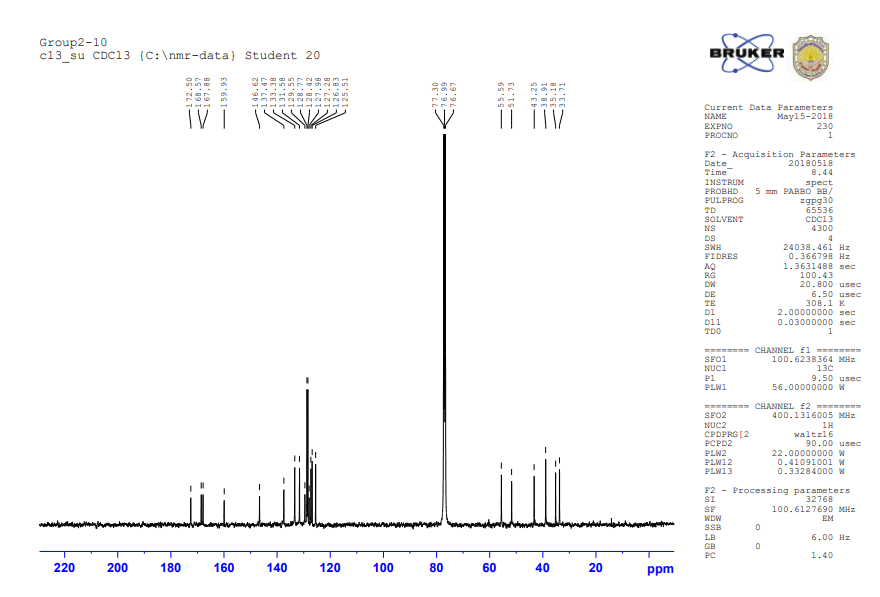
Figure S42**. The ^13^C-NMR spectrum of compound **11b**

**
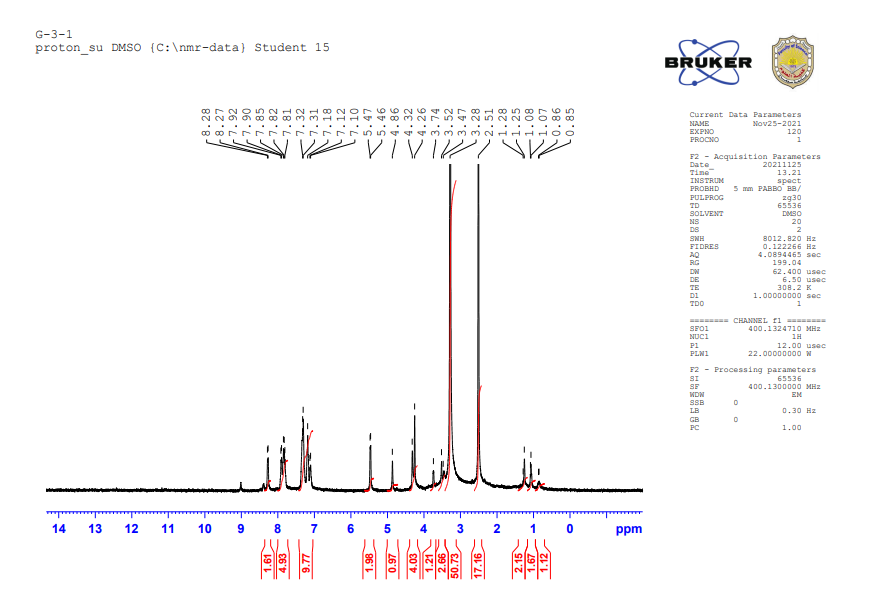
Figure S43**. The ^1^H-NMR spectrum of compound **12a**

**Figure S44**. The ^1^H-NMR spectrum of compound **12b**

**
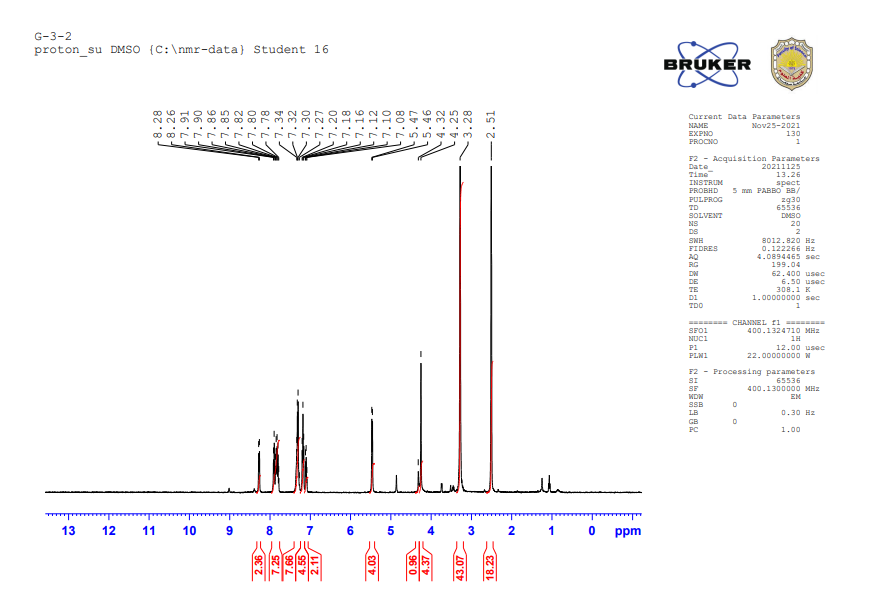
**

**Figure S45**. The ^1^H-NMR spectrum of compound **12c**

**
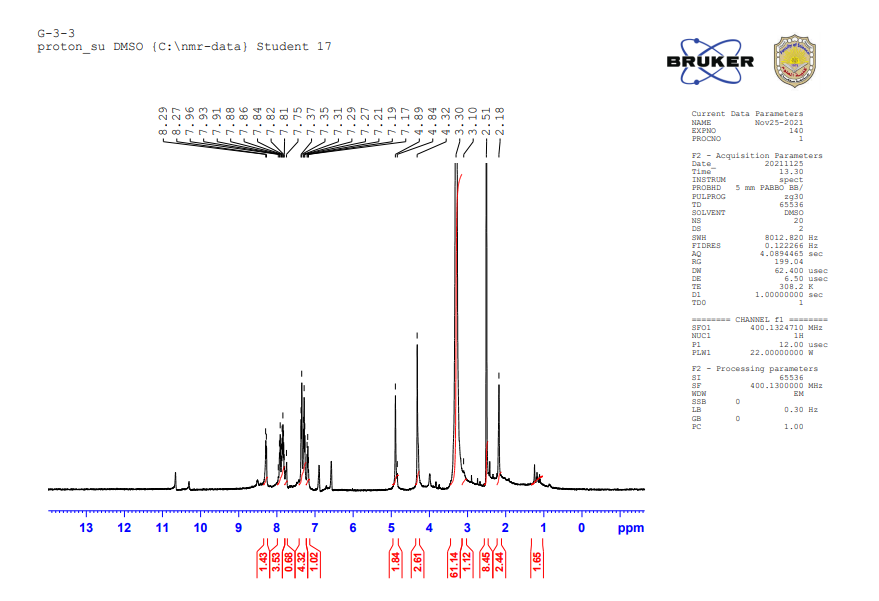
**

**
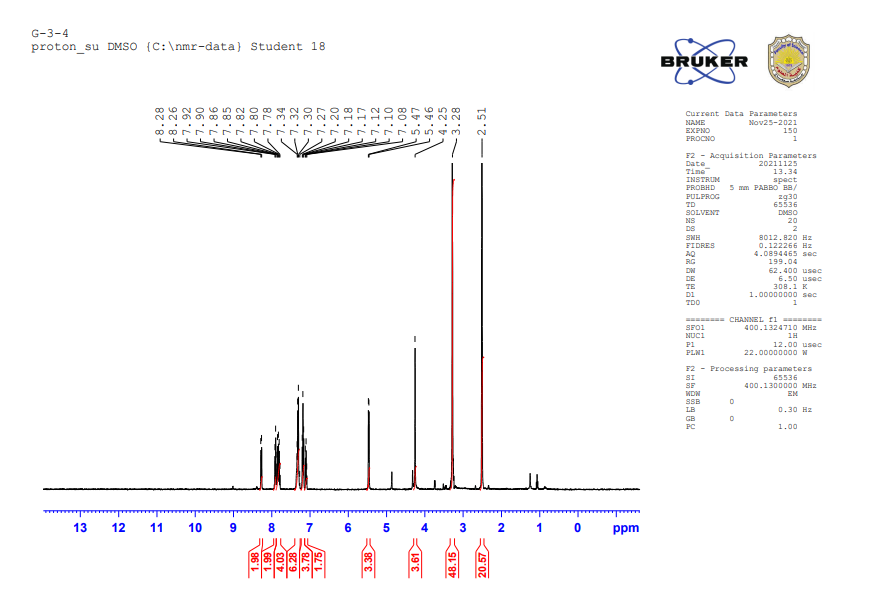
Figure S46**. The ^1^H-NMR spectrum of compound **12d**

**Figure S47**. The ^13^C-NMR spectrum of compound **12d**

**
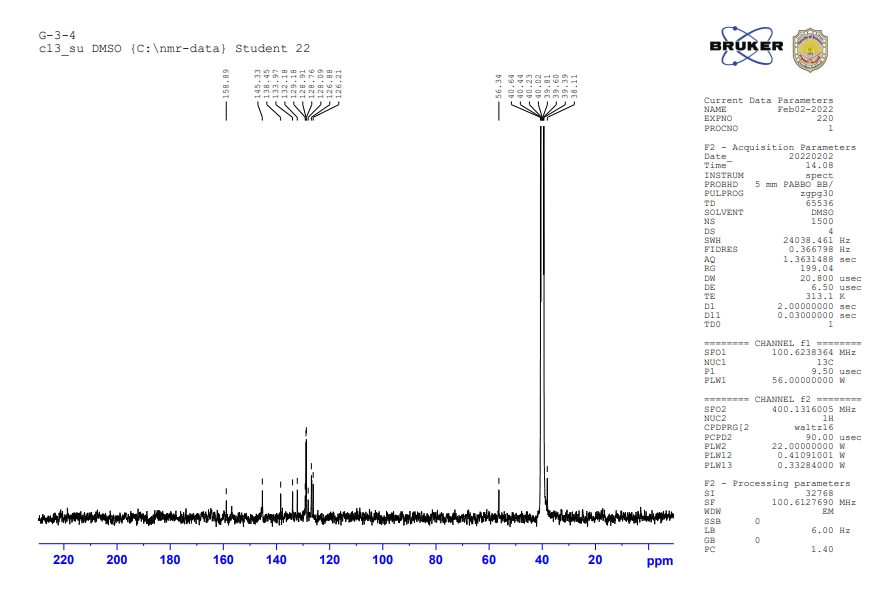
**

**
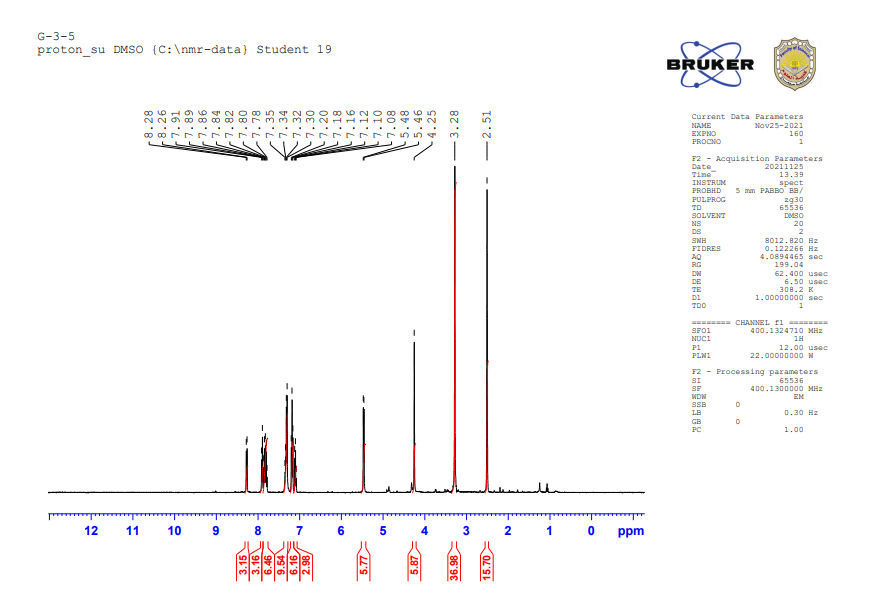
Figure S48**. The ^1^H-NMR spectrum of compound **12e**
